# Unveiling Iso- and Aniso-Hydric Disparities in Grapevine—A Reanalysis by Transcriptome Portrayal Machine Learning

**DOI:** 10.3390/plants13172501

**Published:** 2024-09-06

**Authors:** Tomas Konecny, Armine Asatryan, Maria Nikoghosyan, Hans Binder

**Affiliations:** 1 Armenian Bioinformatics Institute, Yerevan 0014, Armenia; armine.asatryan@abi.am (A.A.); maria.nikoghosyan@abi.am (M.N.); binder@izbi.uni-leipzig.de (H.B.); 2 Interdisciplinary Centre for Bioinformatics, University of Leipzig, 04107 Leipzig, Germany; 3 Group of Plant Genomics, Institute of Molecular Biology, National Academy of Sciences of Armenia, Yerevan 0014, Armenia; 4 Bioinformatics Group, Institute of Molecular Biology, National Academy of Sciences of Armenia, Yerevan 0014, Armenia

**Keywords:** grapevine, climate changes, water(-deficient) stress, transcriptome portrayal, SOM machine learning, stilbenoid biosynthesis, thiamine biosynthesis

## Abstract

Mechanisms underlying grapevine responses to water(-deficient) stress (WS) are crucial for viticulture amid escalating climate change challenges. Reanalysis of previous transcriptome data uncovered disparities among isohydric and anisohydric grapevine cultivars in managing water scarcity. By using a self-organizing map (SOM) transcriptome portrayal, we elucidate specific gene expression trajectories, shedding light on the dynamic interplay of transcriptional programs as stress duration progresses. Functional annotation reveals key pathways involved in drought response, pinpointing potential targets for enhancing drought resilience in grapevine cultivation. Our results indicate distinct gene expression responses, with the isohydric cultivar favoring plant growth and possibly stilbenoid synthesis, while the anisohydric cultivar engages more in stress response and water management mechanisms. Notably, prolonged WS leads to converging stress responses in both cultivars, particularly through the activation of chaperones for stress mitigation. These findings underscore the importance of understanding cultivar-specific WS responses to develop sustainable viticultural strategies in the face of changing climate.

## 1. Introduction

*Vitis vinifera*, being a cornerstone in the global wine industry, faces unprecedented challenges in the era of climate change. Among these challenges, drought stands out as one of the pivotal factors causing water(-deficient) stress (WS), influencing grape ripening and overall fruit quality. As global temperatures rise and precipitation patterns become increasingly unpredictable, the study of WS in *Vitis vinifera* has become paramount for ensuring the resilience of vineyards and the sustainability of wine production.

Current climate change scenarios have brought about shifts in weather patterns, leading to prolonged periods of water scarcity and extreme climatic events. These changes pose a significant threat to viticulture, impacting grapevine physiology, grape quality, and wine production. WS, characterized by a deficiency in water availability relative to the plant’s demand, triggers a cascade of physiological responses in *Vitis vinifera*, affecting its growth, photosynthesis, and metabolic processes [1,2,3,4]. Notably, grapevines have evolved two distinct mechanisms, isohydric and anisohydric, to navigate the complex interplay between water availability and demand [5,6,7,8,9]. Isohydric cultivars regulate water potential by actively adjusting stomatal conductance, conserving water during limited availability, and prioritizing water status regulation over continuous carbon assimilation. Conversely, anisohydric cultivars maintain sustained carbon assimilation even under declining water availability, with a more permissive control over stomatal conductance [10,11,12]. While promoting growth and productivity, this strategy exposes grapevines to higher risks of hydraulic failure under severe water deficit situations [9,13,14].

The investigation of these isohydric and anisohydric mechanisms gains significance in deciphering the adaptive strategies of *Vitis vinifera* to varying water conditions [15,16,17]. The importance of such research is heightened by the need to develop resilient grape cultivars or drought-resistant rootstocks and implement sustainable viticultural practices in response to the changing climate.

Even though both isohydric and anisohydric mechanisms in different grapevine cultivars are well-known and studied at the transcriptomics level [15], current advancements in the field of artificial intelligence (AI) and machine learning (ML) and their applications in analyzing molecular biology data offer exciting possibilities. AI- and ML-powered analyses, particularly through techniques like self-organizing maps (SOM), have the potential to unlock a deeper understanding of the underlying mechanisms and biological pathways that occur at specific time points during stress treatments relevant to viticulture [18]. SOM is a type of artificial neural network that can visualize high-dimensional data like gene expression profiles. By analyzing transcriptomic data using SOM, hidden patterns and relationships between genes can be identified and visualized, enhancing understanding of the underlying mechanisms governing grapevine responses to abiotic stress conditions caused, for instance, by cold temperatures or by water limitation.

A previous study [15] delved into how grapevine transcriptomes respond to WS at the molecular level. The studied isohydric cultivar (‘Montepulciano’) exhibited a faster transcriptome response after WS imposition, with rapid modulation of genes related to abscisic acid (ABA), a stress response hormone, and quicker expression of heat-shock protein (HSP) genes. On the other hand, an anisohydric cultivar (‘Sangiovese’) displayed early and robust induction of reactive oxygen species (ROS)-scavenging enzymes, molecular chaperones, and abiotic-stress-related genes in response to deficiency of water. While this research provides valuable insights, it is important to recognize that the genes identified by significance analysis of microarrays (SAM) represent a portion of the grapevine’s response to WS [19]. Here, we apply SOM omics portrayal to these data to analyze the transcriptomic response of isohydric or anisohydric grapevines under water deficit conditions. SOM transcriptomics portrayal was developed to extract and interpret complex patterns from multidimensional transcriptomic data [20], and it has been applied recently to cold stress acting on *Vitis vinifera* and, particularly, to describe the transcriptomics trajectories of the grapevine plant under changing environmental conditions [18]. Our aim is to compare trajectories associated with drought response in the isohydric and anisohydric grapevine categories. We show that isohydric MP initially maintains a muted transcriptional response, prioritizing transcripts for normal growth (phloem development) before potentially shifting towards activation of stress response processes for survival. Conversely, anisohydric SG displays a rapid transcriptional response, initially focusing on stress alleviation (ketoreductase, arogenate) and water management (lignin catabolism, chitinase activity) before upregulating thiamin biosynthesis, a pathway potentially linked to broader abiotic stress tolerance. Despite their distinct genetic backgrounds related to water-use efficiency, both cultivars eventually converge toward shared responses under prolonged drought conditions.

## 2. Results

### 2.1. WS Influences Gene Expression Trajectories in Distinct Cultivars

**Figure 1:**
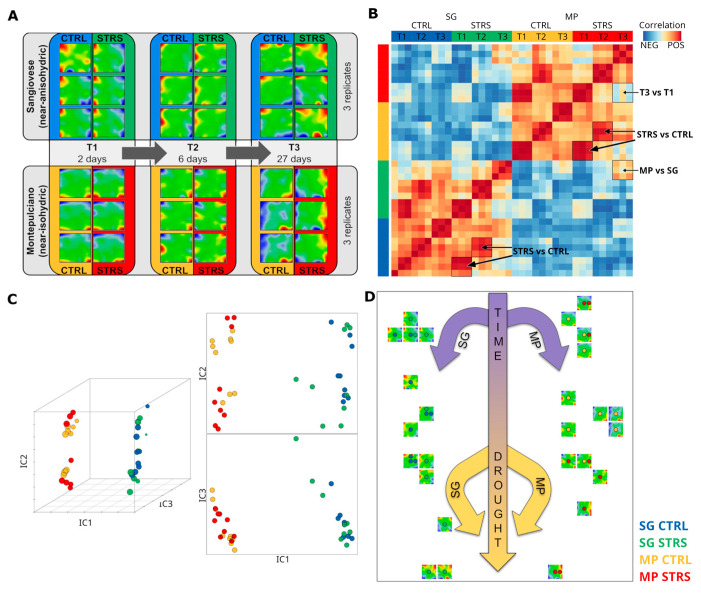
Transcriptome portrayal of the WS experiment reveals distinct trajectories for isohydric (MP) and anisohydric (SG) cultivars. (**A**) SOM transcriptome portraits of all samples studied. (**B**) The pairwise correlation map of the SOM portraits indicates two distinct clusters for the two cultivars, correlation squares along the diagonal due to the replicates, and off-diagonal correlations between different time points and stress-induced effects in both cultivars (see arrows). (**C**) The independent component plot of the portraits shows linear trajectories along the IC2 axis of both cultivars. IC1, IC2, and IC3 denote the first three independent components. (**D**) Sample SOM represents a two-dimensional presentation of the trajectories: Transcriptome trajectories separate due to isohydric and anisohydric cultivars in the horizontal direction and develop with time vertically with an additional increment due to WS. Dots inside each “Sample SOM” represent samples. The color code of samples is indicated in the bottom right corner.

“Drought” stresses the plants due to a hydric deficiency, i.e., the effect of water reserve (e.g., soil water deficit) defined as the difference between the maximum and the actual water availability (field capacity). The experimental setup of Dal Santo and colleagues modeled such (deficient) WS conditions to study physiological responses based on transcriptomic data [15]. They achieved it by comparing one isohydric and one anisohydric cultivar under well-watered and water-stressed conditions as a time series of three measurements in three biological replicates. Our SOM method provides “portrait” images of each of those 36 measurements, visualizing their individual transcriptome landscapes in terms of red overexpression and blue underexpression “spots” (Figure 1A). Note that SOM portrayal methods provide individual images of each sample, which visualize the expression landscape of nearly 30,000 single genes using a color code from blue (low expression) to dark red (high expression) for “spot”-like clusters of co-regulated genes. All images apply the same coordinate system of gene distribution, meaning that they can be directly compared and used for downstream analyses such as similarity and trajectory analysis as well as function mining by means of gene set enrichment analysis (see [20] for a detailed description of the method). Our focus remains on studying changing expression patterns under progressing WS. For an overview, we generated a pairwise correlation heatmap between all portraits. It revealed that the data separated between the two cultivars in terms of two distinct clusters of samples with similar expression patterns evident as dark-red squares reflecting positive correlations between them (Figure 1B). The small red quadrants along the diagonal refer to the similarities of the replicates, as expected. Interestingly, off-diagonal correlations were also observed referring to similarities between STRS and CTRL conditions at T1 and T2, between the two cultivars at T3, as well as to dissimilarities between time points T1 and T3 under WS conditions (see red and blue areas in Figure 1B, respectively). Independent component analysis (ICA) transforms the correlation matrix into a spatial distribution of the samples in the coordinate system of the first three independent components (IC1–IC3). Interestingly, it indicates linear trajectories for each of the cultivars (MP versus SG separated along component IC1) progressing with time (T1 to T3), mostly along IC2 and showing an extra increment under WS (Figure 1C). The so-called “Sample SOM” (Figure 1D) projects these relations into two dimensions. It also shows the two parallel temporal trajectories slightly converging at T3, suggesting an offset between the WS transcriptomic mechanisms of both cultivars and similar stress response mechanisms over time acting in MP and SG.

In summary, SOM portrayal visualizes the expression data of grapevine genes as sample-specific, “individual” images, which enables their visual inspection and comparison. This analysis reveals two different transcriptome clusters for the selected isohydric and anisohydric cultivars, giving rise to two virtually parallel trajectories in sample space, which are indicative of a basic difference of their transcriptomes but similar time courses of their transcriptomes under WS conditions.

### 2.2. Different Water Management Strategies Regulate Similar Genes under Lasting WS

**Figure 2:**
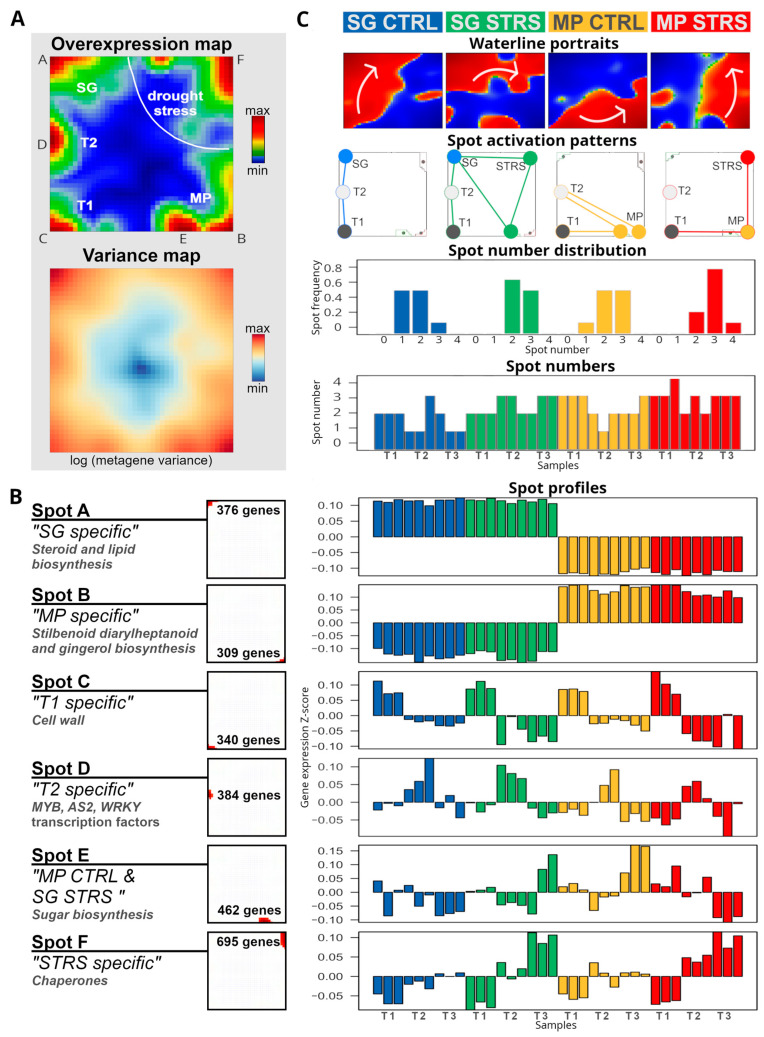
Transcriptome dynamics under WS in isohydric (MP) and anisohydric (SG) water management. (**A**) The regions of characteristic overexpression as red areas labeled A–F (see “Overexpression map”) agree with regions of highest expression variance (see “Variance map”). (**B**) Spots usually contain a few hundred genes of different functional contexts (left). Expression profiles of the spots across all conditions reveal characteristic courses of transcriptomic co-regulation (right). (**C**) Transcriptome dynamics under WS is characterized by waterline portraits revealing different stress trajectories for SG and MP (gray arrows), time, as well as by spot activation patterns (spots jointly activated in the portraits are connected by lines at the time points indicated). The number of jointly expressed spots increases under WS, thus indicating a more complex transcriptomic pattern compared to the controls (see text).

To better understand the changing gene expression patterns behind the observed trajectories, we analyzed the SOM portraits in more detail. The SOM algorithm clusters genes with correlated expression profiles across the time courses together into red “spot-like” modules in the portraits, which were summarized into one overview “Overexpression map” (Figure 2A, part above; for a detailed description of the SOM portrayal method see the Materials and Methods section and references cited therein). Overall, we identified six such modules labeled with capital letters A–F. They collect the most variant genes, and contain around 300 to 600 genes each (Figure 2A,B; lists of genes in the spots are given in Table A1). The expression profiles of the spots divide into characteristic time courses of both cultivars, namely: genes upregulated permanently in SG and downregulated in MP (spot A); the antagonistic profile (spot B); genes activated in both cultivars at T1 (spot C), T2 (spot D), or T3, however differently expressed between SG and MP (spot E); and, finally, genes upregulated at T3 in both cultivars (spot F, see Figure 2B).

Gene set overrepresentation analysis of the gene lists of each of the spots provides a first glimpse at their functional background (Figure 2B). For example, the genes in spot A, differentially upregulated in SG compared with MP, are related to steroid and lipid biosynthesis, while genes in spot B, differentially upregulated in MP compared with SG, are related to stilbenoid, diarylheptanoid, and gingerol biosynthesis. These different functions of the transcriptional programs thus indicate the basic differences between both cultivars at the gene expression level. Temporal progression is driven in both cultivars by the activation of genes related to cell wall pathogen-defensive functions (T1, spot C); different transcription factors such as MYB, WRKY, and AS2 (T2, spot D); sugar biosynthesis (T3, spot E); and chaperones under long-lasting WS (T3, spot F). These factors act as master regulators, turning on genes involved in various drought tolerance mechanisms [21]. MYB transcription factors are known to regulate genes for osmoprotectant synthesis, secondary metabolite production, and WS responses [22,23,24,25,26,27]. WRKY factors are involved in signaling pathways, hormonal routes, defense responses, antioxidant production, and regulation of other stress-responsive genes [28]. AS2 (ASYMMETRIC LEAVES2) plays a co-opted role in stomatal regulation and water use efficiency, with a key role in flat symmetric leaf formation [29].

To better identify subtle changes in expression patterns in the SOM portraits, we generated mean waterline portraits averaged over all time points referring to CTRL and STRS conditions of MP and SG, respectively (Figure 2C, part above). They highlight regions of up- and downregulated genes in red and blue, respectively, and thus enable us to visualize subtle modulations of gene expression levels more clearly than the standard portraits shown in Figure 1. Two different trajectories for SG and MP in gene space under WS can be identified, pointing in clock- and counterclockwise directions, respectively, and both converging in the right upper corner around spot F (see arrows).

The spots identified in Figure 2A are activated in various combinations, depending on the experimental conditions. This results in diverse configurations of the gene expression landscapes, as depicted by the networks of spot activation (Figure 2C, second row from the top, co-expressed spots are linked by lines). Note that the activation pattern follows the arrows in the waterline portraits. For SG, the spot modules progressively activate along the left and upper edges of the map. For MP, the spot modules activate along the lower and right edges. The activation patterns of SG and MP converge in spot F under lasting WS at T3. The number of jointly activated spots is slightly larger in SG compared to MP and increases under stress, thus reflecting a more complex transcriptional pattern (Figure 2C, the two rows from the bottom). This increased number of jointly activated spots reflects a higher complexity of the underlying transcriptional programs, which slightly increases under lasting WS.

Hence, SOM portrayal complements the two different trajectories for the isohydric and anisohydric cultivars observed in the sample space (see previous subsection) by trajectories in the gene expression space, which enables identification of jointly and differently activated transcriptional programs as a function of the cultivars, the WS conditions, and the time points.

### 2.3. Topology of Transcriptional Programs under Progressing WS

**Figure 3:**
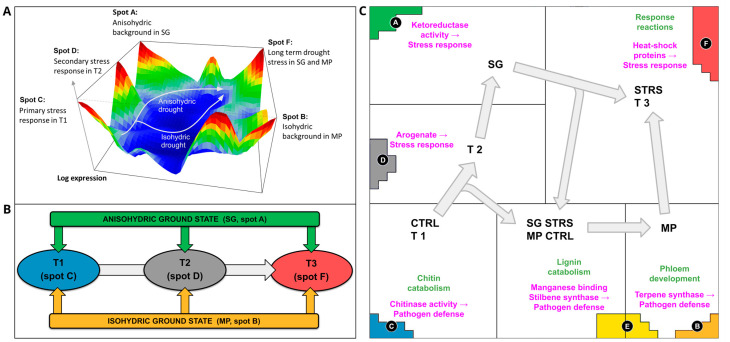
Topology of expression trajectories under WS. (**A**) The stress trajectories in the three-dimensional expression landscape. (**B**) Schematic spot activation along the T1-T2-T3 trajectory. (**C**) Gene ontology enrichment in spots A–F (biological processes in green, molecular functions in pink text).

As shown in the previous section, our spot activation analysis revealed two different trajectories in the gene expression landscape pointing from T1 to T3 in the clockwise and counterclockwise direction for the SG and MP cultivars, respectively (Figure 3A). We asked about the transcriptional programs associated with the different sets of genes taken from the spots to better understand the functional background of the observed WS dynamics. Spot A and B include genes antagonistically activated in SG and MP, respectively, forming a sort of background expression for the anisohydric and isohydric cultivars, respectively. The progression from T1 to T3 along IC2 in ICA analysis (see Figure 1C above) is driven by the sequential activation of spots C, D, and F, respectively (see the scheme in Figure 3B). Hence, the topology of gene activations as visualized by the SG- and MP-trajectories in the three-dimensional gene expression landscape (Figure 3A) and, alternatively, by the spot activation workflow in Figure 3B rationalizes the common and different properties of the isohydric and anisohydric cultivar dynamics under WS.

Figure 3C provides a functional map associating the genes in the spots with the underlying biological functions using gene set enrichment analysis. The anisohydric SG, exhibiting open stomata and being more prone to water deficiency stress, remains consistently predisposed to WS. The genes specifically activated in SG accumulate in spot A (Figure 3C). They associate with functions such as steroid and lipid biosynthesis. We found an enrichment of ketoreductase activity (GO:0045703). Hence, the upregulation of ketoreductases observed in SG background (spot A) can serve as an initial indication of stress accompanied by the plant response to reactive oxygen species [30,31].

In contrast to SG, the isohydric cultivar MP is better adapted to sustain water levels during drought, which is demonstrated by the upregulation of genes associated with plant normal growth, development, transportation, and signaling pathways (see spots B and E in Figure 3C). For instance, some of these genes are linked to stilbenoid, diarylheptanoid, and gingerol biosynthesis. Additionally, there is also GO enrichment in phloem development (GO:0010088), promoting the development of phloem cells, and terpene synthase activity (GO:0010333) that plays a role in plant defense response to environment and pathogens [32]. 

At the first time point T1, genes in spot C (Figure 3C) upregulate in both MP and SG cultivars. They are enriched by gene sets involved in chitin catabolism (GO:0006032) and cell wall development. A strengthened cell wall (chitin and lignin) is a common defense strategy against drought [33,34], as well as pathogens [35]. The second time point, T2, upregulated genes in spot D (Figure 3C), which is occupied by genes coding transcription factors (MYB, AS2, WRKY). The transcriptional reprogramming mediated by MYB, AS2, and WRKY transcription factors orchestrates broader regulatory networks involved in stress signaling, defense responses, and developmental adjustments reacting to prolonged water shortage. The MYB transcription factors, for example, regulate the secondary metabolism, hormone signaling, and stress responses, contributing to drought tolerance mechanisms [27,36,37]. AS2 transcription factors participate in leaf morphology and physiology which optimizes the efficiency of water management under drought conditions [29,38]. WRKY transcription factors, the integral components of many vines’ stress signaling pathways, coordinate biological processes associated with stress tolerance, such as scavenging of ROS, osmotic regulation, and ABA signaling [39]. There is also an enrichment in GO molecular activity of arogenate dehydrogenase NADP+ (GO:0033730) in spot D. Arogenate is a precursor of phenylalanine that plays a significant role in plant abiotic stress responses [40,41,42]. Hence, with the increasing time under drought, more comprehensive genes are involved in the WS response.

Spot E is upregulated at T3 in SG (under STRS) and MP (under CTRL), thus inducing a certain asymmetry between both trajectories. The gene list of this spot is enriched by sugar biosynthesis genes. For anisohydric cultivars such as SG, sugar serves as an osmoprotectant that maintains cellular hydration under lasting WS [43]. Additionally, this spot is enriched by genes related to lignin catabolism (GO:0046274), playing a role in water management, manganese ion binding (GO:0030145), and trihydroxystilbene synthesis (GO:0050350), considered to be important for pathogen defense [44,45,46]. Hence, osmoprotection and pathogen response become activated in the anisohydric cultivar under lasting WS, while the isohydric cultivar activates these functions under water excess.

STRS-specific spot F (Figure 3C) contains genes enriched by heat-shock/protein-complex oligomerization proteins (GO:0051259) from the family HSP20 acting as molecular chaperones. Such chaperones help plants fight against WS by facilitating protein folding, assembly, stabilization, and degradation [47]. Despite the differences in transcriptomes between SG and MP in T1/T2, water deficiency induces a similar response in prolonged exposure (T3).

Taken together, different “ground state” functions related to lipid and steroid biosynthesis in SG and plant growth functions in isohydric MP give rise to their specific trajectories, which both progress under WS sequentially by activation of transcription factors such as MYB, AS2, and WRKY, ABA-signaling, and arogenate-mediated stress response, and after lasting WS finally express HSP coding genes. An asymmetry in WS dynamics between SG and MP is related to osmoprotection by sugar biosynthesis and lignin catabolism in SG STRS and in MP under CTRL conditions, respectively.

### 2.4. Isohydric Strategy Is Characterized by Biosynthesis of Stilbenoid and Its Derivatives

**Figure 4:**
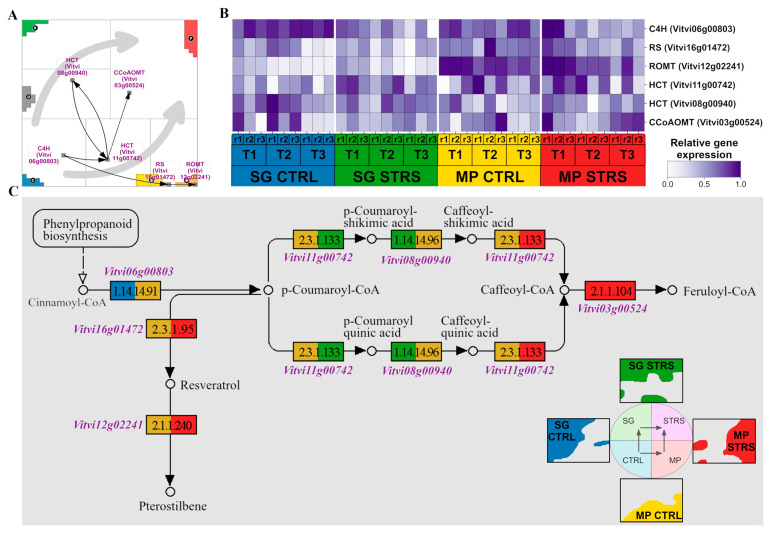
Stilbenoid, diarylheptanoid, and gingerol biosynthetic pathway. (**A**) Genes of the pathway (downstream flow visualized by black arrows) are along the two STRS trajectories (gray arrows), indicating their condition-specific activation. (**B**) The heatmap shows the expression changes in response to WS. (**C**) KEGG pathway with gene color code derived from their positions in the SOM portrait indicated by the colored waterline portraits of SG CTRL and STRS and MP CTRL and STRS (see the lower right part).

Our analysis of gene expression patterns revealed that the isohydric grape cultivar, known for its water-conserving strategies, appears to activate stilbenoid biosynthesis as a marker of drought tolerance, partly in its ground state. Genes related to stilbenoid biosynthesis distribute in a characteristic fashion in the expression landscape with a certain asymmetry towards the MP trajectory (Figure 4A). Although located mostly outside the spots due to the smaller variance in their expression, they can be assigned to different conditions, as discussed in the previous subsection. The heatmap in Figure 4B explicitly provides their expression profiles, revealing their time-dependent activation under the different conditions, which shows their biased upregulation in the isohydric MP cultivar.

For a closer look, we mapped these activation patterns on the stilbenoid KEGG pathway topology, where the gene boxes are colored according to the condition-dependent activation (Figure 4C). CINNAMATE 4-HYDROXYLASE (C4H), encoded by the gene *Vitvi06g00803*, is an important precursor enzyme for stilbenoid biosynthesis. The gene was found to be upregulated in both SG and MP under optimal CTRL water supply conditions, as indicated by the blue and dark yellow colors, respectively. C4H catalyzes the hydroxylation of trans-cinnamic acid to produce p-coumaric acid, an important intermediate in the biosynthesis of various compounds, including stilbenoids [48]. Next, there is an enzyme HYDROXYCINNAMOYL-CoA:SHIKIMATE/QUINATE TRANSFERASE (HCT–HST/HQT), whose instability allows for various reactions to occur [49,50]. This enzyme is encoded by the genes *Vitvi11g00742* and *Vitvi08g00940*, which exhibited interesting expression dynamics. *Vitvi11g00742* showed higher expression in MP CTRL during the initial reaction, whereas it was upregulated in SG STRS. This enzyme mediates the formation of p-Coumaroyl shikimic acid and p-Coumaroyl quinic acid [50,51]. Subsequent reactions showed differential expression patterns, with *Vitvi11g00742* shifting its overexpression entirely to MP STRS. The enzyme CAFFEOYL-COA O-METHYLTRANSFERASE (CCoAOMT), encoded by gene *Vitvi03g00524*, emerged as a dominant player in (isohydric) MP STRS as well. It is known to respond to water deficiency stress [52]. Other downstream enzymes involved in the synthesis of stilbenoids, namely RESVERATROL SYNTHASE (RS) and RESVERATROL O-METHYLTRANSFERASE (ROMT), were found to be exclusively overexpressed in MP STRS conditions. The RS enzyme, encoded by *Vitvi16g01472*, catalyzes the condensation of malonyl-CoA with p-Coumaroyl-CoA to form resveratrol [53], while the enzyme ROMT (encoded by *Vitvi12g02241*) methylates the resveratrol to produce a pterostilbene [54]. All these genes exhibited higher expression levels in MP under both control and stress conditions, suggesting a potential role for resveratrol and pterostilbene in response to water deficiency stress in MP in agreement with the topology of WS transcriptome dynamics (see Figure 3C).

In summary, the detailed mapping of gene expression patterns onto the stilbenoid pathway elucidated the overall asymmetric role of various members of this pathway in MP’s and SG’s distinct responses to WS, where, notably, resveratrol biosynthetic genes were uniquely upregulated in the isohydric MP.

### 2.5. Biosynthesis of Thiamine Is Promoted under WS Conditions

**Figure 5:**
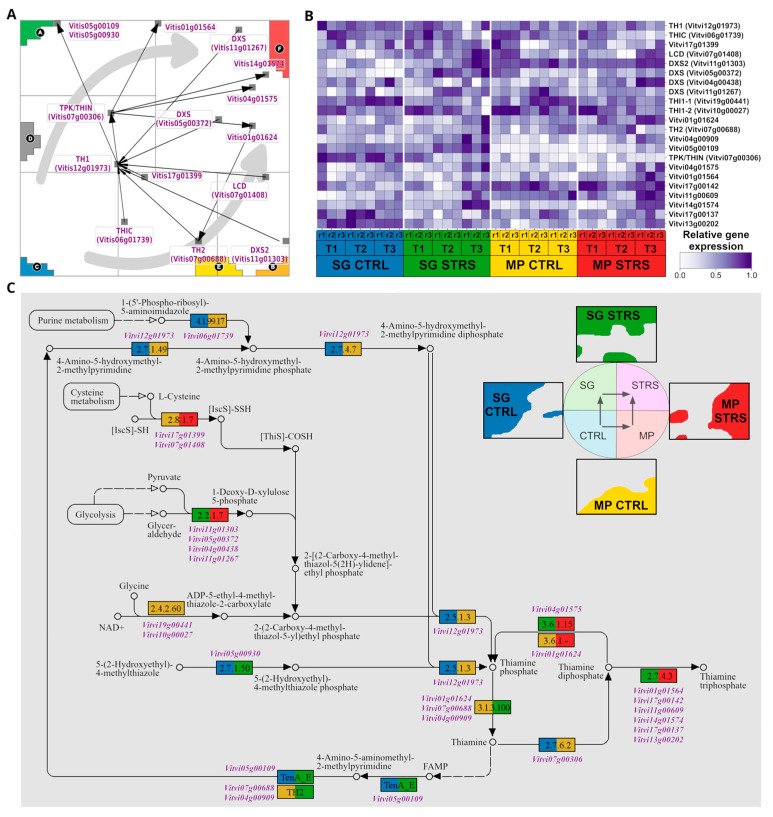
Thiamine biosynthetic pathway. (**A**) Map of the pathway (downstream flow visualized by black arrows). Genes accumulate in areas related to stress response, and the two STRS trajectories are shown by gray arrows. (**B**) Heatmap depicting the thiamine biosynthetic pathway gene expression in response to WS. (**C**) KEGG pathway with gene color code derived from their positions in the SOM portrait indicated by the colored waterline portraits of SG CTRL and STRS and MP CTRL and STRS (see the upper right part).

Next, we aimed to elucidate the thiamine biosynthesis pathway in grapevines (Figure 5). This pathway was shown to react under conditions of low-temperature stress in *Vitis vinifera* [18] and was assumed to play an important role also under WS conditions [55]. The “gene map” of the thiamine biosynthesis pathway genes, as well as their expression heatmap, reveal the gene activation across the whole SOM landscape except the T1-specific area (Figure 5A,B). As for the pathway of biosynthesis of stilbenoid and its derivatives, we transferred the gene expression profiles into the KEGG pathway topology (Figure 5C). Under control conditions, both MP and SG cultivars exhibited nearly stable expression of genes involved in thiamine biosynthesis. These genes encode enzyme isoforms, including THIAZOLE (TH1), PYRIMIDINE SYNTHETASE (THIC), and THIAMINE PYROPHOSPHOKINASE (TPK/THIN), which plays a role in synthesizing the active form of thiamine. In plants, three forms of thiamine are physiologically active: free thiamine, thiamine monophosphate, and thiamine pyrophosphate. The Enzyme Commission number is depicted by the colored (blue, yellow) rectangles in the pathway diagram (see Figure 5C). Notably, the expression of these genes in both cultivars is similar also under WS, indicating a basal level of thiamine biosynthesis activity in grapevine plants.

Under WS, both cultivars exhibited the upregulation of structural genes, encoding isoforms of the enzyme 1-DEOXY-D-XYLULOSE-5-PHOSPHATE SYNTHASE (DXS). DXS is associated with the precursor production for both thiamine and isoprenoid biosynthesis [56], particularly DXP production from glycolysis. Isoprenoids, which have a positive correlation with the level of DXP transcript [57], play crucial roles in plant stress tolerance mechanisms [58]. Other studies have shown that the overexpression of DXS leads to increased chlorophyll and carotenoid content [59]. Furthermore, under the stress conditions, a cluster of genes was upregulated at the final stage of the pathway. Products of these genes convert the active form of thiamine, thiamine diphosphate (TPP), either to thiamine triphosphate (*Vitvi01g001564*, *Vitvi17g00142*, *Vitvi11g00609*, *Vitvi17g00137*, *Vitvi14g01574*, *Vitvi13g00202*) or thiamine monophosphate (*Vitvi04g01575*).

The grapevine gene *Vitvi11g01303*, encoding a probable enzyme 1-DEOXY-D-XYLULOSE-5-PHOSPHATE SYNTHASE2 (DXS2), exhibits upregulation in SG STRS compared to SG CTRL and even higher overexpression in MP STRS (Figure 5B). The presence of multiple DXS isoforms with distinct expression patterns in both cultivars suggests a potentially intricate regulatory mechanism governing DXP production in response to WS. The third precursor gene in thiamine biosynthesis, *Vitvi07g01408*, producing an enzyme L-CYSTEINE DESULFURASE 1 (LCD), provides the essential sulfur atom for thiazole precursor synthesis [60]. It was notably overexpressed under the WS compared to control conditions.

In summary, our analysis shows the various activations of thiamine biosynthesis genes under WS, from the initiation of precursor production to the final conversion of thiamine di-/tri- phosphate. This comprehensive understanding offers valuable insights into the distinct responses of MP and SG cultivars, potentially influencing their resilience to prolonged water deficit.

### 2.6. Exploring Differentially Expressed Genes Taken from the Original Study

**Figure A1:**
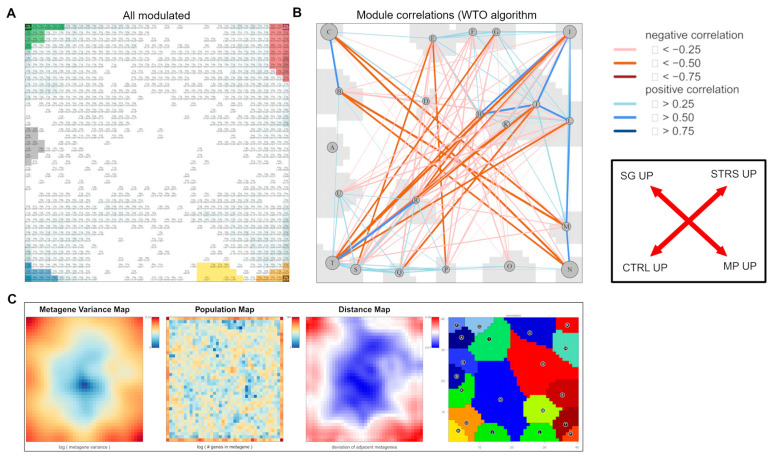
Covariance and population structure of the SOM expression landscape. (**A**) The 5947 genes significantly modulated in leaves under our experimental conditions were taken from the SAM analysis in [15] and mapped into our SOM. Occupied metagenes (pixels) are marked in greyish. They refer mostly to metagenes of high and moderate variance of gene expression (see part C). (**B**) The covariance structure of the SOM was estimated by calculating a weighted topological overlap (WTO) network between the spot modules [65]. It reveals strong anticorrelations (w < 0) between the two cultivars (SG vs. MP) and between the two conditions (STRS vs. CTRL; see the scheme on the right). It thus assigns the “modulated genes” (part A) to up- and downregulation under the different conditions. Note also that the SOM decomposes the “modulated genes” into clusters of co-regulated genes called spot modules. (**C**) The variance map of the metagenes reveals a variance gradient of gene expression from the center of the SOM (blue) towards its edges (brown). The population map color codes the number of genes per metagene from high (red) to low (blue). The gene density changes across the SOM. Also, the Euclidean distances between neighboring metagenes are variant and “amplify” regions of increased gene density. The K-means map provides a space-filling segmentation of the SOM, enabling it to consider any region for downstream analysis, such as function mining.

**Figure A2:**
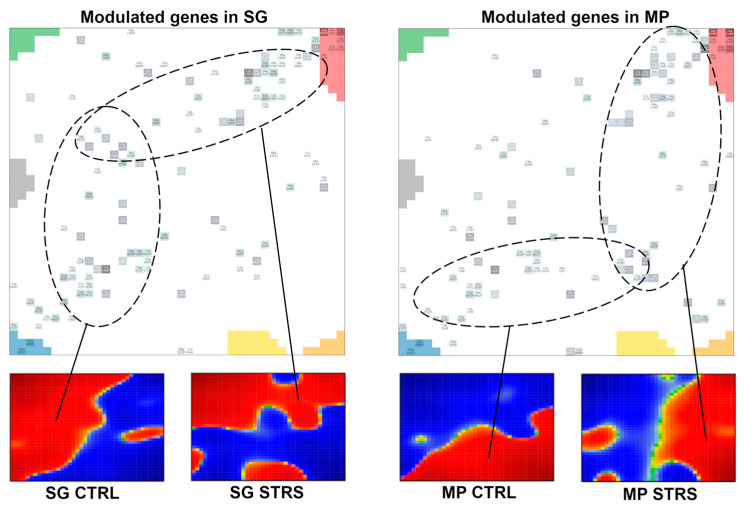
Genes specifically modulated in the accessions were taken from supporting data [15] and compared to the respective waterline portraits. Genes modulated in SG and MP accumulate in the overexpressed areas in SG and MP, respectively.

Mapping of specifically modulated genes from the previous study [15] into our SOM landscape supports our results regarding a set of about 6000 highly differentially expressed genes (Figure A1). SOM portrayal, however, disentangles and visualizes in detail their covariance structure not evident from the original list. Notably, SOM portrayal distributes these genes across a transcriptome landscape, obeying the topology of transcriptomic co-regulation networks. Specifically, modulated genes in SG and MP fit into the WS trajectories extracted from the SOM analysis and visualized by the upregulated regions of the waterline portraits, as expected (Figure A2). However, SOM analysis more clearly disentangles the modulated genes into distinct modules of coregulation with distinct functional impact and, moreover, extracts higher genes of stronger variance compared with the significance analysis of microarrays (SAM) and ANOVA algorithms applied in the original paper [15] and the references cited therein. SAM should be used with caution because of the limitations of the method, particularly in overrating threshold settings and variance corrections [61].

**Figure A3:**
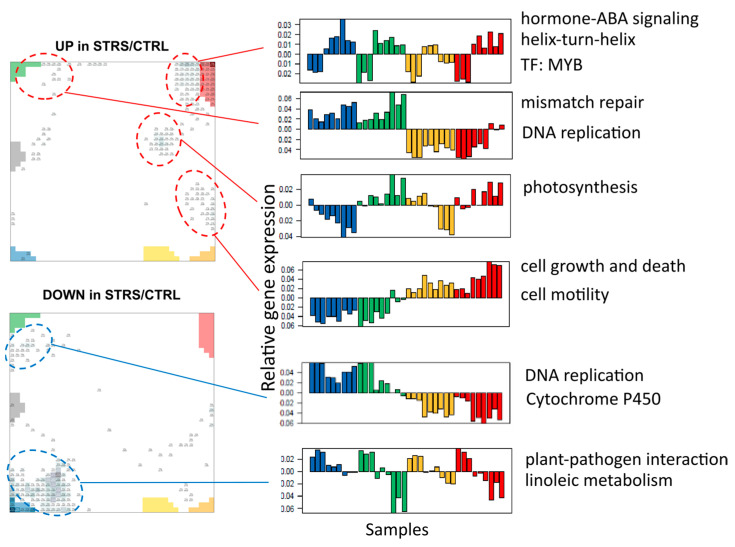
Genes modulated specifically under stress conditions. The cutoffs defining upregulated (UP in STRS/CTRL) and downregulated (DOWN in STRS/CTRL) genes were taken from [15] (fold change > 2 and top 20% variant genes) and mapped into the SOM. Clusters of genes accumulate in different areas of the SOM in and near the spots. The respective profiles are shown on the right, together with the respective biological functions. As expected, the profiles confirm the UP/DOWN under STRS conditions, showing the respective changes, especially under lasting stress at T3. The profiles also show that the expression changes are about one order of magnitude smaller compared with that observed in the spots. The bars are color-coded as in the other figures.

**Figure A4:**
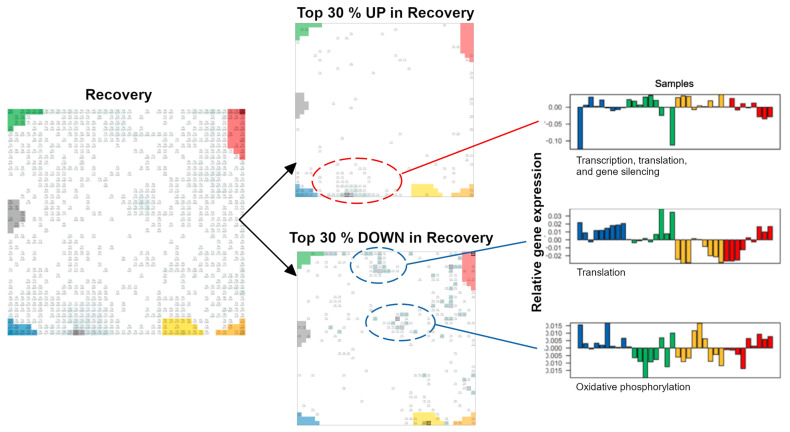
Genes specifically modulated under recovery from WS. Recovery conditions were achieved by re-watering the plants 46 days after the onset of WS, and the post-recovery plant material was collected 70 days after the onset of WS (see Materials and Methods in [15]). Upregulated (UP) and downregulated (DOWN) genes and their respective profiles are shown separately. The profiles refer to the stress experiment. Recovery virtually reverses expression changes observed for lasting stress (STRS at T3).

Up- and downregulated genes, due to the WS, extract additional modules of co-regulated genes with different functional impacts, such as photosynthesis and DNA repair (Figure A3). Finally, we also considered genes differentially expressed under a “recovery from the WS” (i.e., re-watering 70 days after the onset of the WS), which indicates the partial reversal of the gene expression changes caused by prolonged WS (Figure A4). 

Our SOM analysis demonstrates highly similar differential gene expression patterns for the genes previously reported. SOM considers genes of higher variance, which extends the range of the functional impact of WS dynamics. Note also that our SOM landscape reflects expression patterns without pre-selecting case vs. control conditions (e.g., STRS vs. CTRL, SG vs. MP), which were addressed in downstream analyses as applied above. It thus provides a sort of holistic modular transcriptomic map that considers the cultivar, conditions, and time points independently, which is required for estimating the trajectories of WS in the SOM landscape and not explicitly available using the SAM and ANOVA approaches.

## 3. Discussion

In this study, we reanalyzed whole-genome transcriptional microarray data of *Vitis vinifera* leaves [15] by applying the SOM clustering algorithm [62,63], which has previously been successfully employed in analyzing gene expression data on grapevine cultivars under low-temperature stress conditions [18]. We are motivated for this study by a series of key advantages of SOM machine learning, such as high-resolution clustering into function-relevant modules of co-regulated genes, intuitive visualization of whole transcriptome landscapes with single-sample resolution (SOM portrayal), the combination of dimension and redundancy reduction with whole data processing, statistical power of feature selection and tracking expression trajectories in a topology-aware transcriptome landscape, which, overall, enabled the detailed analysis of gene expression changes over the stress period (2, 6, and 27 days), to compare both isohydric and anisohydric strategies of two selected cultivars against WS. For a detailed description of the method and its strength in different applications, we refer to our previous work [20,64,65,66]. Here we continue our studies on aspects of genomic regulation of grapevine physiology suffering from abiotic stress after considering cold temperature stress [18].

The isohydric and anisohydric cultivars show distinct physiological and morphological changes under WS [5,6,15,67,68,69]. To understand the underlying gene regulation of drought-responsive processes at the transcriptome and pathway levels in two grapevine genotypes differing in water management strategy, we performed SOM portrayal and WS trajectory analysis in sample and gene space in combination with detailed knowledge mining of the patterns of the identified coregulated genes. The divergence of transcriptional programs between SG and MP in response to WS exemplifies the two different stress-response strategies of these grapevine cultivars.

In accordance with the previous findings in the original reference publication [15], our research identified the activation of genes coding for chaperones and heat-shock proteins in response to prolonged WS. Chaperones and heat-shock proteins are crucial components of the cell’s machinery for protein folding. They assist in the proper folding of proteins and prevent the aggregation of misfolded proteins, which can be detrimental to cellular function and survival. Under normal conditions, proteins fold into their functional, three-dimensional structures with the help of chaperones. However, under stress conditions like drought, the normal protein folding process can be disrupted, leading to the accumulation of misfolded or unfolded proteins. Hence, the activation of genes coding for chaperones and heat-shock proteins is a critical component of the plant’s response to long-lasting WS.

Our trajectory analysis based on module activation adds a novel, more comprehensive view of WS dynamics in the anisohydric and isohydric cultivars not addressed by Dal Santo and colleagues [15]. SOM portrayal reveals that during the initial stages of water deprivation, both vine cultivars adjust the cell wall composition and structure that might be necessary to cope with reduced water availability. This suggests that it starts with physical reinforcement of the cell wall, followed by broader stress signaling and adjustment, and finally, adaptation for long-term survival through compatible solutes and protein stability. This could help the grapevine to maintain cell integrity under the pressure of water deficit, reduce water loss by creating a tighter barrier, and enhance resistance to potential secondary stresses like pathogens that might exploit drought-weakened plants. The activation of specific transcription factors like MYB, WRKY, and AS2 signifies a shift towards a broader stress response. We speculate that the WS-driven upregulation of steroid and lipid biosynthesis, specifically in anisohydric SG, strengthens cell membranes, while ketoreductases help detoxify harmful substances. This response also involves the production of phytohormones, like ABA and antioxidants, to protect cells from damage. 

On the other hand, the isohydric MP prevails in water conservation and stomatal regulation, which diminishes water loss under WS. The stilbenoids, diarylheptanoids, and gingerols possess antioxidant properties so they could mitigate oxidative stress induced by drought. The isohydric vine may allocate resources towards a synthesis of such compounds to bolster the defense mechanisms against drought-induced oxidative stress and enhance tolerance to water deficit. 

Moreover, our analysis indicates fast activation of stilbenoid biosynthesis genes (*C4H*, *RS*, *ROMT*, promiscuous *HCT,* and *CCoAOMT*) in the isohydric grape cultivar under WS. Particularly, the increased expression of resveratrol biosynthesis genes in MP (compared to SG) implies that wines derived from this cultivar may accumulate higher resveratrol content than those from the SG cultivar under WS conditions.

In our previous work, we identified the upregulation of thiamine biosynthetic genes in low- and extremely low-temperature stress [18], which motivated us to analyze this pathway under WS. Notably, recent studies have highlighted the significance of thiamine in the plant response to WS [70,71], demonstrating significant upregulation of *THI1* and *THIC* genes under drought and dry air humidity stresses [72,73]. Our findings support the hypothesis that the production of thiamine is not only a cold acclimation stress-responsive but also a WS-responsive compound in both iso- and aniso-hydric vine cultivars. Here, we provided more elaborate insights into the cascade of gene activation underlying stress response mechanisms in grapevine.

The focus on stilbenoid and thiamine pathways is, although justified by the data and motivated by our previous work, somewhat arbitrary, but it illustrates the potency of the transcriptomic data for extracting WS responses at the pathway level. Although their phenotypes assign them isohydric and anisohydric characteristics, differential gene expression between them does not necessarily directly relate to molecular causes of the different water management strategies. Future investigations should generalize the results by extending the number of vine accessions in similar experimental and analysis settings.

In conclusion, the dynamics of biological processes in both studied cultivars under WS and control conditions might shed light on further vine research. Experimental studies are needed to fully understand these mechanisms and their potential applications in improving drought resistance, which has significant implications for vine production, particularly in regions where water availability is a major constraint. In the future, this research might help to explore the possibility of manipulating biochemical pathways to enhance drought tolerance in other vine cultivars, securing viticulture in the face of changing climate. Overall, our findings underscore the genomic diversity and different adaptability of these cultivars to changing environments.

## 4. Materials and Methods

### 4.1. Expression Data and Treatment Conditions

The gene expression data were downloaded from the supporting dataset (GEO: GSE70670; http://www.ncbi.nlm.nih.gov/geo/query/acc.cgi?token=ovqbgaqedfaxjqn&acc=GSE70670; accessed on 3 February 2024) provided by Dal Santo and colleagues [15]. It encompasses normalized gene expression data (Nimblescan v2.5 software-calculated Robust Multiarray Average/RMA-normalized signal intensities) from leaves (L) of two grapevine (*Vitis vinifera* L.) cultivars, ‘Montepulciano’—clone R7 (here referred to as MP) and ‘Sangiovese’—clone VCR30 (here referred to as SG), possessing isohydric and anisohydric characteristics, respectively. For a detailed description of the experimental set-up, we refer to the original publication [15]. In short, both cultivars were grafted onto 1103 Paulsen rootstock, and 8-year-old, well-maintained, greenhouse-potted vines were used in the experiment. Two groups were established for each cultivar: a water-stressed group (STRS) that received 40% of maximum water availability relative to the soil field capacity of 30.2% [(vol water/vol soil) × 100], and a well-watered control group (CTRL) that received 90% of maximum water availability. The samples were collected for the analysis of transcriptome at three time points following the initiation of WS: 2, 6, and 27 days (referred to as T1, T2, and T3, respectively). These time points represent different stages of drought stress, ranging from the initial onset of WS to prolonged exposure to the stress. The experiment included three biological replicates (r1, r2, and r3) for each condition in both cultivars. Dal Santo and colleagues complemented transcriptomic measurement with phenotypic data, particularly leaf physiological and leaf biochemical parameters. We refer to the original publication for details [15].

### 4.2. Self-Organizing Map

The SOM portrayal algorithm was employed to analyze the preprocessed gene expression data using the oposSOM software (version 2.2.5), as described previously [18]. A total of 36 samples were used as input, containing expression values for 28,521 genes. These represent 81.18% of the 35,134 annotated coding genes of *Vitis vinifera* that were found in the Ensembl database [74,75] on the 16th of May, 2023. The SOM was configured to have 1600 neurons (also called metagenes, characterizing the mean transcriptional profile of the genes collected in the respective neuron), arranged in a quadratic lattice with dimensions 40 × 40. During training, the SOM distributed the gene expression values across the neurons/metagenes, effectively reducing the high-dimensional data into a 2-dimensional representation. The gene expression profiles of each treatment condition, including control and stressed samples for both cultivars, were mapped onto the SOM grid. The resulting SOM, obtained for each treatment condition, was visualized as an expression “portrait”. To enable the visual interpretation of these SOM “portraits”, a tertiary color code was used to color each metagene, ranging from red through green to blue, corresponding to high to low gene expression. As the metagenes that are located close to each other include genes with similar expression profiles, we can define red and blue spots in the SOM “portraits”. Red spots signify transcript upregulation, while blue spots indicate transcript downregulation, deviating more than the upper or lower quartile in a positive or negative direction, respectively.

Alternatively, we used “waterline” profiles, indicating expression values above and below the mean in red and blue, respectively, to better visualize slight changes in the transcriptome patterns. Statistics of the spot expression in terms of jointly activated spots in the individual portraits, spot number frequencies, and mean numbers of spots per portrait provide details of the topology of the activated transcriptome under the different treatment conditions. For a comparison of differentially expressed genes published in the previous study [15] with our SOM results, we mapped the selected marker genes into our SOM expression landscape to show their distribution (see Figure A1, Figure A2, Figure A3 and Figure A4). The sample similarity analysis uses a pairwise correlation map, independent component analysis, and sample SOM presentations. Details of the methods used and their applications to different use cases were described previously [20,64,65,66] and are available in the R-package oposSOM [62].

### 4.3. Functional Annotation

KEGG [76] and VitisNet [77] databases were used to functionally annotate genes for the gene set analysis, and PANTHER (version 18.0; [78]) was employed with default settings for the gene ontology (GO) enrichment analysis (for details see [18]). Genes from the KEGG pathways mentioned in this work were mapped onto SOM overexpression spots.

## Data Availability

Expression data were taken from a previous study [15]. The R-object of our SOM analysis is deposited online in the Leipzig Health Atlas with the accession number 611 (LHA:611; https://www.health-atlas.de/data_files/611 accessed on 26 July 2024).

## Appendix A

**Table A1:** Lists of genes in the SOM spots A–F. Number of genes per each spot is in parentheses.

A (376 Genes)	B (309 Genes)	C (340 Genes)	D (384 Genes)	E (462 Genes)	F (695 Genes)
*Vitvi17g00237*	*Vitvi10g02124*	*Vitvi15g01586*	*Vitvi04g02074*	*Vitvi14g02553*	*Vitvi13g00164*
*Vitvi16g01025*	*Vitvi08g01847*	*Vitvi18g00424*	*Vitvi04g00410*	*Vitvi09g01725*	*Vitvi06g01513*
*Vitvi01g00437*	*Vitvi10g02194*	*Vitvi10g00667*	*Vitvi18g02126*	*Vitvi13g02480*	*Vitvi13g00169*
*Vitvi01g00580*	*Vitvi18g02451*	*Vitvi15g01587*	*Vitvi18g00833*	*Vitvi19g02069*	*Vitvi12g00573*
*Vitvi01g01028*	*Vitvi05g01951*	*Vitvi10g01684*	*Vitvi01g01736*	*Vitvi17g01664*	*Vitvi07g02152*
*Vitvi19g00388*	*Vitvi10g02121*	*Vitvi04g00501*	*Vitvi19g00456*	*Vitvi07g02304*	*Vitvi14g01193*
*Vitvi18g02718*	*Vitvi07g00973*	*Vitvi05g01760*	*Vitvi04g02122*	*Vitvi10g00867*	*Vitvi19g00618*
*Vitvi14g01657*	*Vitvi17g01737*	*Vitvi13g01250*	*Vitvi19g01484*	*Vitvi09g01261*	*Vitvi13g00166*
*Vitvi14g02664*	*Vitvi17g01732*	*Vitvi11g00016*	*Vitvi02g01517*	*Vitvi04g02117*	*Vitvi13g00471*
*Vitvi15g01691*	*Vitvi10g02195*	*Vitvi01g01917*	*Vitvi18g00596*	*Vitvi17g01693*	*Vitvi13g00162*
*Vitvi16g01878*	*Vitvi08g01380*	*Vitvi15g00970*	*Vitvi05g01287*	*Vitvi16g01659*	*Vitvi04g01974*
*Vitvi04g00827*	*Vitvi13g00925*	*Vitvi07g02119*	*Vitvi19g01674*	*Vitvi16g00770*	*Vitvi07g00055*
*Vitvi07g01789*	*Vitvi17g01736*	*Vitvi06g00268*	*Vitvi10g01493*	*Vitvi04g01870*	*Vitvi05g01933*
*Vitvi03g01162*	*Vitvi10g02181*	*Vitvi07g02123*	*Vitvi08g01548*	*Vitvi18g02894*	*Vitvi06g00526*
*Vitvi18g00871*	*Vitvi04g01325*	*Vitvi16g00941*	*Vitvi16g00027*	*Vitvi01g02297*	*Vitvi18g01438*
*Vitvi17g01541*	*Vitvi10g02190*	*Vitvi18g01937*	*Vitvi07g02288*	*Vitvi07g02725*	*Vitvi09g01282*
*Vitvi18g02157*	*Vitvi12g00224*	*Vitvi15g00714*	*Vitvi03g01582*	*Vitvi14g02863*	*Vitvi10g00034*
*Vitvi16g02001*	*Vitvi18g02293*	*Vitvi01g01934*	*Vitvi15g01364*	*Vitvi14g01112*	*Vitvi18g02423*
*Vitvi02g01350*	*Vitvi11g00460*	*Vitvi02g01404*	*Vitvi13g02086*	*Vitvi18g02584*	*Vitvi10g01778*
*Vitvi04g01352*	*Vitvi04g02105*	*Vitvi07g01844*	*Vitvi13g02044*	*Vitvi19g01302*	*Vitvi07g00259*
*Vitvi13g01356*	*Vitvi18g02289*	*Vitvi18g01263*	*Vitvi10g01807*	*Vitvi14g01082*	*Vitvi13g02077*
*Vitvi05g01256*	*Vitvi06g01097*	*Vitvi18g02446*	*Vitvi10g00859*	*Vitvi14g01106*	*Vitvi08g00731*
*Vitvi08g01052*	*Vitvi00g00339*	*Vitvi04g00760*	*Vitvi10g02180*	*Vitvi14g01080*	*Vitvi18g01070*
*Vitvi19g01504*	*Vitvi16g01302*	*Vitvi15g01582*	*Vitvi15g00736*	*Vitvi16g01613*	*Vitvi02g01447*
*Vitvi12g02444*	*Vitvi18g03097*	*Vitvi02g01403*	*Vitvi14g03039*	*Vitvi17g01674*	*Vitvi07g00455*
*Vitvi18g03185*	*Vitvi19g00216*	*Vitvi10g00742*	*Vitvi17g00315*	*Vitvi15g01363*	*Vitvi01g02273*
*Vitvi12g02069*	*Vitvi12g00602*	*Vitvi05g00011*	*Vitvi19g01322*	*Vitvi14g02865*	*Vitvi18g02559*
*Vitvi19g01984*	*Vitvi04g01233*	*Vitvi18g02287*	*Vitvi14g02789*	*Vitvi19g02154*	*Vitvi10g00403*
*Vitvi01g00350*	*Vitvi04g01375*	*Vitvi03g01621*	*Vitvi18g01927*	*Vitvi14g02533*	*Vitvi19g02371*
*Vitvi14g02925*	*Vitvi14g00297*	*Vitvi14g00500*	*Vitvi04g00160*	*Vitvi16g02162*	*Vitvi06g01575*
*Vitvi19g00107*	*Vitvi13g01320*	*Vitvi06g01604*	*Vitvi08g02076*	*Vitvi08g01978*	*Vitvi02g00020*
*Vitvi01g01982*	*Vitvi02g00783*	*Vitvi06g01601*	*Vitvi13g00109*	*Vitvi14g01075*	*Vitvi14g03078*
*Vitvi12g00927*	*Vitvi08g02249*	*Vitvi02g00393*	*Vitvi16g01641*	*Vitvi14g02548*	*Vitvi05g00139*
*Vitvi18g00717*	*Vitvi12g02165*	*Vitvi14g01381*	*Vitvi18g03208*	*Vitvi14g01096*	*Vitvi19g00381*
*Vitvi17g00609*	*Vitvi04g01940*	*Vitvi03g00708*	*Vitvi09g01460*	*Vitvi16g00772*	*Vitvi05g00681*
*Vitvi15g00005*	*Vitvi06g01100*	*Vitvi07g01697*	*Vitvi14g03080*	*Vitvi10g01046*	*Vitvi06g01746*
*Vitvi00g01279*	*Vitvi05g02283*	*Vitvi05g02248*	*Vitvi08g01941*	*Vitvi06g01301*	*Vitvi12g00301*
*Vitvi11g00526*	*Vitvi10g02113*	*Vitvi18g02727*	*Vitvi07g02115*	*Vitvi10g01626*	*Vitvi16g00205*
*Vitvi02g01806*	*Vitvi07g02402*	*Vitvi11g01674*	*Vitvi12g02456*	*Vitvi12g02239*	*Vitvi11g01450*
*Vitvi03g01891*	*Vitvi09g01214*	*Vitvi18g00979*	*Vitvi01g01387*	*Vitvi10g00869*	*Vitvi09g00246*
*Vitvi10g02223*	*Vitvi05g00669*	*Vitvi18g02592*	*Vitvi07g02146*	*Vitvi09g00878*	*Vitvi11g01333*
*Vitvi10g00001*	*Vitvi13g01889*	*Vitvi19g00094*	*Vitvi08g02329*	*Vitvi18g03390*	*Vitvi11g00251*
*Vitvi12g02448*	*Vitvi18g02463*	*Vitvi12g02244*	*Vitvi08g01303*	*Vitvi13g02426*	*Vitvi10g00459*
*Vitvi08g01404*	*Vitvi15g01669*	*Vitvi11g01491*	*Vitvi19g02218*	*Vitvi13g01559*	*Vitvi14g01951*
*Vitvi08g01617*	*Vitvi02g01037*	*Vitvi05g01577*	*Vitvi04g02233*	*Vitvi18g03068*	*Vitvi05g00461*
*Vitvi14g01127*	*Vitvi13g01404*	*Vitvi14g01868*	*Vitvi14g02756*	*Vitvi18g02935*	*Vitvi16g01985*
*Vitvi13g01180*	*Vitvi15g01663*	*Vitvi07g02735*	*Vitvi18g01565*	*Vitvi16g01469*	*Vitvi12g02469*
*Vitvi11g01251*	*Vitvi01g00555*	*Vitvi09g01536*	*Vitvi04g00988*	*Vitvi00g02157*	*Vitvi03g00119*
*Vitvi14g02469*	*Vitvi16g02010*	*Vitvi14g01488*	*Vitvi12g01676*	*Vitvi18g02870*	*Vitvi04g02187*
*Vitvi05g02198*	*Vitvi10g02119*	*Vitvi15g00844*	*Vitvi17g00662*	*Vitvi14g02864*	*Vitvi19g02000*
*Vitvi17g00788*	*Vitvi18g02631*	*Vitvi02g00391*	*Vitvi08g00199*	*Vitvi18g03332*	*Vitvi00g02173*
*Vitvi14g02722*	*Vitvi10g02301*	*Vitvi06g01515*	*Vitvi08g02328*	*Vitvi01g01614*	*Vitvi12g00028*
*Vitvi03g01584*	*Vitvi05g01940*	*Vitvi07g00665*	*Vitvi13g02029*	*Vitvi09g00908*	*Vitvi18g02906*
*Vitvi01g00605*	*Vitvi14g02575*	*Vitvi14g02913*	*Vitvi18g03011*	*Vitvi14g01113*	*Vitvi08g02117*
*Vitvi03g01890*	*Vitvi16g01449*	*Vitvi08g02147*	*Vitvi10g01983*	*Vitvi13g00931*	*Vitvi07g00293*
*Vitvi04g02192*	*Vitvi17g00972*	*Vitvi01g01921*	*Vitvi02g01439*	*Vitvi16g01474*	*Vitvi19g01760*
*Vitvi17g00639*	*Vitvi18g02464*	*Vitvi09g00264*	*Vitvi05g00716*	*Vitvi04g02121*	*Vitvi08g02393*
*Vitvi03g01603*	*Vitvi08g01848*	*Vitvi12g02565*	*Vitvi09g00524*	*Vitvi17g00592*	*Vitvi02g00543*
*Vitvi18g02061*	*Vitvi10g01613*	*Vitvi12g02718*	*Vitvi08g02343*	*Vitvi13g02424*	*Vitvi05g01837*
*Vitvi18g02716*	*Vitvi18g02273*	*Vitvi07g02114*	*Vitvi19g02019*	*Vitvi08g00035*	*Vitvi04g01794*
*Vitvi17g00405*	*Vitvi10g02174*	*Vitvi18g03084*	*Vitvi14g02445*	*Vitvi18g01019*	*Vitvi04g01920*
*Vitvi14g01222*	*Vitvi02g01850*	*Vitvi02g01440*	*Vitvi18g02586*	*Vitvi16g01481*	*Vitvi19g02268*
*Vitvi05g01439*	*Vitvi12g02275*	*Vitvi08g00768*	*Vitvi18g00164*	*Vitvi18g02881*	*Vitvi02g00264*
*Vitvi00g02337*	*Vitvi12g02241*	*Vitvi11g00726*	*Vitvi14g00940*	*Vitvi12g02350*	*Vitvi14g01726*
*Vitvi01g00065*	*Vitvi18g03265*	*Vitvi01g00710*	*Vitvi14g02691*	*Vitvi09g02069*	*Vitvi07g02229*
*Vitvi03g01887*	*Vitvi12g02245*	*Vitvi07g02604*	*Vitvi12g02386*	*Vitvi16g01457*	*Vitvi19g01791*
*Vitvi08g00942*	*Vitvi07g02390*	*Vitvi07g02650*	*Vitvi09g00304*	*Vitvi00g02261*	*Vitvi14g01942*
*Vitvi13g02451*	*Vitvi12g02243*	*Vitvi08g01291*	*Vitvi04g00013*	*Vitvi12g02338*	*Vitvi02g00162*
*Vitvi12g01836*	*Vitvi12g02274*	*Vitvi11g00467*	*Vitvi09g01984*	*Vitvi12g02340*	*Vitvi08g01656*
*Vitvi18g02722*	*Vitvi18g03098*	*Vitvi14g01815*	*Vitvi18g02521*	*Vitvi16g01702*	*Vitvi13g01839*
*Vitvi12g00596*	*Vitvi11g01701*	*Vitvi14g00461*	*Vitvi07g01519*	*Vitvi16g00133*	*Vitvi05g01839*
*Vitvi01g00697*	*Vitvi01g00532*	*Vitvi06g00931*	*Vitvi19g00604*	*Vitvi15g01406*	*Vitvi07g00117*
*Vitvi07g01990*	*Vitvi11g01037*	*Vitvi05g00710*	*Vitvi07g02322*	*Vitvi12g01852*	*Vitvi01g00329*
*Vitvi04g02196*	*Vitvi02g01649*	*Vitvi19g00321*	*Vitvi12g02387*	*Vitvi09g00920*	*Vitvi08g02274*
*Vitvi14g01149*	*Vitvi13g00701*	*Vitvi14g02930*	*Vitvi15g01372*	*Vitvi11g00126*	*Vitvi16g01022*
*Vitvi12g02742*	*Vitvi08g01650*	*Vitvi18g00675*	*Vitvi04g02129*	*Vitvi13g01566*	*Vitvi13g02070*
*Vitvi19g00656*	*Vitvi07g01308*	*Vitvi09g00233*	*Vitvi10g02222*	*Vitvi19g02190*	*Vitvi07g01296*
*Vitvi12g00036*	*Vitvi12g02292*	*Vitvi14g00483*	*Vitvi07g02949*	*Vitvi18g02746*	*Vitvi14g01354*
*Vitvi03g00406*	*Vitvi05g01934*	*Vitvi01g00784*	*Vitvi06g00040*	*Vitvi09g00907*	*Vitvi06g01360*
*Vitvi12g00478*	*Vitvi12g00243*	*Vitvi15g00314*	*Vitvi04g00467*	*Vitvi16g00761*	*Vitvi07g00380*
*Vitvi12g01984*	*Vitvi12g02285*	*Vitvi16g01213*	*Vitvi17g00895*	*Vitvi06g01812*	*Vitvi05g00857*
*Vitvi12g02569*	*Vitvi19g02111*	*Vitvi04g00442*	*Vitvi03g01521*	*Vitvi07g02369*	*Vitvi13g02192*
*Vitvi14g01952*	*Vitvi09g01872*	*Vitvi13g00509*	*Vitvi13g00546*	*Vitvi09g01782*	*Vitvi09g01904*
*Vitvi16g01697*	*Vitvi10g01656*	*Vitvi14g01977*	*Vitvi14g02916*	*Vitvi16g00251*	*Vitvi08g02143*
*Vitvi09g01145*	*Vitvi01g00574*	*Vitvi06g01597*	*Vitvi13g01737*	*Vitvi13g01564*	*Vitvi10g00330*
*Vitvi06g00714*	*Vitvi17g01739*	*Vitvi02g01661*	*Vitvi16g01122*	*Vitvi14g02773*	*Vitvi18g01510*
*Vitvi07g00130*	*Vitvi03g01777*	*Vitvi06g00790*	*Vitvi07g03080*	*Vitvi16g01464*	*Vitvi00g02264*
*Vitvi08g01333*	*Vitvi07g02231*	*Vitvi18g00967*	*Vitvi09g00700*	*Vitvi16g00991*	*Vitvi08g01543*
*Vitvi05g01151*	*Vitvi06g01577*	*Vitvi04g02241*	*Vitvi06g00894*	*Vitvi18g03026*	*Vitvi16g01144*
*Vitvi17g00175*	*Vitvi18g02508*	*Vitvi18g00956*	*Vitvi14g02449*	*Vitvi09g01663*	*Vitvi19g02274*
*Vitvi15g01651*	*Vitvi18g00290*	*Vitvi18g00476*	*Vitvi14g00958*	*Vitvi03g01749*	*Vitvi13g00490*
*Vitvi19g02029*	*Vitvi04g01241*	*Vitvi08g01335*	*Vitvi01g02264*	*Vitvi17g01665*	*Vitvi13g00688*
*Vitvi14g02515*	*Vitvi03g01885*	*Vitvi13g00114*	*Vitvi14g01939*	*Vitvi14g02570*	*Vitvi05g00118*
*Vitvi12g02454*	*Vitvi09g01187*	*Vitvi10g01615*	*Vitvi09g00560*	*Vitvi02g01072*	*Vitvi08g02015*
*Vitvi16g02146*	*Vitvi12g02289*	*Vitvi11g01488*	*Vitvi10g01009*	*Vitvi19g01220*	*Vitvi17g00554*
*Vitvi07g00021*	*Vitvi07g01814*	*Vitvi16g01931*	*Vitvi15g01257*	*Vitvi12g02345*	*Vitvi08g00656*
*Vitvi02g00171*	*Vitvi16g02005*	*Vitvi08g00630*	*Vitvi07g00781*	*Vitvi14g00218*	*Vitvi10g00558*
*Vitvi18g01884*	*Vitvi03g01896*	*Vitvi02g01459*	*Vitvi13g00556*	*Vitvi05g01039*	*Vitvi05g01392*
*Vitvi10g01650*	*Vitvi15g01762*	*Vitvi16g01932*	*Vitvi18g02673*	*Vitvi18g02889*	*Vitvi04g01793*
*Vitvi17g01414*	*Vitvi12g02269*	*Vitvi08g01702*	*Vitvi09g00708*	*Vitvi07g02079*	*Vitvi07g00256*
*Vitvi04g01815*	*Vitvi12g01121*	*Vitvi19g00404*	*Vitvi08g01625*	*Vitvi05g02015*	*Vitvi06g00999*
*Vitvi14g03033*	*Vitvi17g01302*	*Vitvi19g00690*	*Vitvi14g02736*	*Vitvi14g02550*	*Vitvi09g00996*
*Vitvi14g01211*	*Vitvi08g01382*	*Vitvi18g01938*	*Vitvi03g01556*	*Vitvi07g02387*	*Vitvi05g00204*
*Vitvi07g00586*	*Vitvi08g02247*	*Vitvi04g01481*	*Vitvi04g02014*	*Vitvi19g01921*	*Vitvi09g00275*
*Vitvi04g00282*	*Vitvi05g02086*	*Vitvi18g01001*	*Vitvi07g01653*	*Vitvi16g01473*	*Vitvi07g03183*
*Vitvi18g01895*	*Vitvi10g02106*	*Vitvi14g01739*	*Vitvi01g00291*	*Vitvi16g01570*	*Vitvi12g00444*
*Vitvi01g00319*	*Vitvi00g01453*	*Vitvi11g00641*	*Vitvi10g01994*	*Vitvi14g02828*	*Vitvi05g01840*
*Vitvi18g02320*	*Vitvi04g01890*	*Vitvi03g01276*	*Vitvi07g00843*	*Vitvi19g00423*	*Vitvi14g01943*
*Vitvi00g00342*	*Vitvi12g00610*	*Vitvi08g01701*	*Vitvi18g02791*	*Vitvi12g02277*	*Vitvi08g01532*
*Vitvi01g01884*	*Vitvi18g00860*	*Vitvi09g00971*	*Vitvi13g00635*	*Vitvi19g02228*	*Vitvi02g00263*
*Vitvi17g01393*	*Vitvi14g02609*	*Vitvi05g00094*	*Vitvi07g01638*	*Vitvi18g01447*	*Vitvi05g00430*
*Vitvi16g01566*	*Vitvi19g00445*	*Vitvi10g01734*	*Vitvi11g00914*	*Vitvi15g01326*	*Vitvi12g00462*
*Vitvi07g01720*	*Vitvi12g02702*	*Vitvi10g00436*	*Vitvi18g01056*	*Vitvi18g01442*	*Vitvi07g02212*
*Vitvi16g00908*	*Vitvi01g02003*	*Vitvi19g02050*	*Vitvi18g00241*	*Vitvi12g02617*	*Vitvi14g00971*
*Vitvi12g02080*	*Vitvi10g02129*	*Vitvi09g01399*	*Vitvi10g00938*	*Vitvi12g02337*	*Vitvi07g00598*
*Vitvi13g00025*	*Vitvi01g00611*	*Vitvi01g00525*	*Vitvi04g00570*	*Vitvi15g01680*	*Vitvi17g00116*
*Vitvi17g00358*	*Vitvi05g02215*	*Vitvi09g01616*	*Vitvi07g02462*	*Vitvi05g02038*	*Vitvi06g00561*
*Vitvi06g01093*	*Vitvi12g02238*	*Vitvi10g02351*	*Vitvi11g01323*	*Vitvi08g02128*	*Vitvi03g00206*
*Vitvi08g01403*	*Vitvi13g01312*	*Vitvi15g01355*	*Vitvi09g01966*	*Vitvi13g02355*	*Vitvi19g02012*
*Vitvi19g01503*	*Vitvi04g01519*	*Vitvi17g00804*	*Vitvi17g00119*	*Vitvi19g01313*	*Vitvi05g01842*
*Vitvi18g03070*	*Vitvi12g02273*	*Vitvi03g00325*	*Vitvi02g01703*	*Vitvi06g01980*	*Vitvi11g00389*
*Vitvi13g00026*	*Vitvi19g00424*	*Vitvi05g00544*	*Vitvi17g01599*	*Vitvi14g02818*	*Vitvi12g02668*
*Vitvi19g00525*	*Vitvi14g02594*	*Vitvi11g00243*	*Vitvi16g01630*	*Vitvi18g03324*	*Vitvi14g00038*
*Vitvi03g00482*	*Vitvi19g01884*	*Vitvi06g00303*	*Vitvi02g00042*	*Vitvi09g00924*	*Vitvi08g01777*
*Vitvi11g00528*	*Vitvi05g01943*	*Vitvi07g02605*	*Vitvi15g00206*	*Vitvi03g00976*	*Vitvi17g00617*
*Vitvi16g02105*	*Vitvi00g01462*	*Vitvi16g01980*	*Vitvi07g01693*	*Vitvi05g01181*	*Vitvi03g00164*
*Vitvi10g00164*	*Vitvi16g01251*	*Vitvi09g01694*	*Vitvi08g01439*	*Vitvi16g00749*	*Vitvi13g00062*
*Vitvi02g00056*	*Vitvi13g00702*	*Vitvi10g00787*	*Vitvi08g01047*	*Vitvi18g03058*	*Vitvi17g00778*
*Vitvi12g00598*	*Vitvi00g01457*	*Vitvi04g00960*	*Vitvi19g02100*	*Vitvi11g01574*	*Vitvi04g01368*
*Vitvi04g02198*	*Vitvi06g00379*	*Vitvi11g01676*	*Vitvi19g02077*	*Vitvi17g01680*	*Vitvi05g01834*
*Vitvi01g00822*	*Vitvi19g01685*	*Vitvi17g01467*	*Vitvi04g01583*	*Vitvi05g01861*	*Vitvi05g01841*
*Vitvi09g00790*	*Vitvi18g03280*	*Vitvi08g01384*	*Vitvi10g00881*	*Vitvi19g01043*	*Vitvi03g01391*
*Vitvi19g00205*	*Vitvi12g02433*	*Vitvi10g01616*	*Vitvi17g00605*	*Vitvi14g02547*	*Vitvi18g01545*
*Vitvi18g00764*	*Vitvi14g00896*	*Vitvi01g00567*	*Vitvi12g00491*	*Vitvi16g01591*	*Vitvi06g00440*
*Vitvi14g00103*	*Vitvi12g00334*	*Vitvi07g03053*	*Vitvi05g01838*	*Vitvi09g00912*	*Vitvi08g00125*
*Vitvi09g01979*	*Vitvi14g02924*	*Vitvi18g02835*	*Vitvi19g01305*	*Vitvi09g00894*	*Vitvi08g01843*
*Vitvi05g02009*	*Vitvi17g01731*	*Vitvi10g02034*	*Vitvi10g01014*	*Vitvi16g01619*	*Vitvi04g00135*
*Vitvi19g01817*	*Vitvi14g02759*	*Vitvi13g00870*	*Vitvi08g02259*	*Vitvi13g01445*	*Vitvi02g00395*
*Vitvi12g00554*	*Vitvi12g02272*	*Vitvi07g01739*	*Vitvi01g01912*	*Vitvi15g01321*	*Vitvi05g02185*
*Vitvi13g01904*	*Vitvi02g01832*	*Vitvi06g00317*	*Vitvi18g02806*	*Vitvi14g02543*	*Vitvi08g02412*
*Vitvi07g00810*	*Vitvi15g00107*	*Vitvi17g01691*	*Vitvi17g01105*	*Vitvi18g03041*	*Vitvi10g00474*
*Vitvi01g00585*	*Vitvi11g00990*	*Vitvi13g01389*	*Vitvi13g02337*	*Vitvi07g02362*	*Vitvi14g00318*
*Vitvi19g01883*	*Vitvi09g01673*	*Vitvi08g01221*	*Vitvi11g00498*	*Vitvi09g01465*	*Vitvi02g00505*
*Vitvi03g01886*	*Vitvi14g03049*	*Vitvi03g00251*	*Vitvi16g00808*	*Vitvi10g01301*	*Vitvi15g00511*
*Vitvi01g00320*	*Vitvi00g01467*	*Vitvi04g00486*	*Vitvi03g01481*	*Vitvi14g02544*	*Vitvi18g01131*
*Vitvi19g01819*	*Vitvi11g01222*	*Vitvi04g00510*	*Vitvi13g01790*	*Vitvi13g02078*	*Vitvi15g00816*
*Vitvi13g02187*	*Vitvi07g02710*	*Vitvi10g00613*	*Vitvi01g00260*	*Vitvi13g02275*	*Vitvi01g00745*
*Vitvi19g00522*	*Vitvi16g01531*	*Vitvi16g00401*	*Vitvi13g01862*	*Vitvi03g01736*	*Vitvi03g00024*
*Vitvi07g03077*	*Vitvi18g02844*	*Vitvi11g00098*	*Vitvi01g02287*	*Vitvi11g01249*	*Vitvi13g02457*
*Vitvi19g00565*	*Vitvi16g00913*	*Vitvi19g00218*	*Vitvi18g03359*	*Vitvi06g01157*	*Vitvi10g01237*
*Vitvi19g01893*	*Vitvi05g01229*	*Vitvi13g00172*	*Vitvi14g01937*	*Vitvi03g01738*	*Vitvi03g01746*
*Vitvi13g02505*	*Vitvi09g00889*	*Vitvi02g00564*	*Vitvi06g01975*	*Vitvi18g02945*	*Vitvi11g00518*
*Vitvi12g00330*	*Vitvi12g02240*	*Vitvi16g00282*	*Vitvi18g01065*	*Vitvi18g03349*	*Vitvi02g01270*
*Vitvi03g01593*	*Vitvi14g01681*	*Vitvi10g01840*	*Vitvi14g01508*	*Vitvi04g01050*	*Vitvi08g01391*
*Vitvi06g00310*	*Vitvi09g01798*	*Vitvi18g01705*	*Vitvi14g02809*	*Vitvi14g03083*	*Vitvi14g00156*
*Vitvi01g00399*	*Vitvi16g01894*	*Vitvi11g00099*	*Vitvi03g01863*	*Vitvi10g01988*	*Vitvi13g02073*
*Vitvi08g01032*	*Vitvi10g02167*	*Vitvi01g01751*	*Vitvi12g02413*	*Vitvi16g02163*	*Vitvi03g00586*
*Vitvi19g00586*	*Vitvi13g01006*	*Vitvi13g01913*	*Vitvi05g02256*	*Vitvi03g01867*	*Vitvi07g00732*
*Vitvi00g02232*	*Vitvi17g01735*	*Vitvi00g01963*	*Vitvi02g01587*	*Vitvi15g01678*	*Vitvi18g00664*
*Vitvi14g02660*	*Vitvi15g01136*	*Vitvi08g01637*	*Vitvi09g00142*	*Vitvi13g02089*	*Vitvi01g00603*
*Vitvi09g00177*	*Vitvi19g02311*	*Vitvi10g01391*	*Vitvi06g00553*	*Vitvi13g01562*	*Vitvi05g00221*
*Vitvi00g02336*	*Vitvi09g01552*	*Vitvi01g00953*	*Vitvi10g00110*	*Vitvi06g01905*	*Vitvi08g01718*
*Vitvi14g00649*	*Vitvi03g01404*	*Vitvi08g01423*	*Vitvi05g00509*	*Vitvi18g03391*	*Vitvi10g00469*
*Vitvi12g02178*	*Vitvi04g00859*	*Vitvi19g00410*	*Vitvi12g00555*	*Vitvi10g00866*	*Vitvi06g01375*
*Vitvi18g03059*	*Vitvi11g00764*	*Vitvi10g01632*	*Vitvi18g02401*	*Vitvi12g00196*	*Vitvi03g00488*
*Vitvi18g02887*	*Vitvi07g02430*	*Vitvi03g01572*	*Vitvi07g01764*	*Vitvi14g00675*	*Vitvi05g00271*
*Vitvi03g01483*	*Vitvi02g00526*	*Vitvi02g00114*	*Vitvi08g02182*	*Vitvi11g01350*	*Vitvi15g01681*
*Vitvi07g01970*	*Vitvi08g01634*	*Vitvi18g02692*	*Vitvi09g01752*	*Vitvi12g00351*	*Vitvi07g00164*
*Vitvi12g02435*	*Vitvi05g01938*	*Vitvi07g01429*	*Vitvi07g01475*	*Vitvi14g02535*	*Vitvi03g00445*
*Vitvi18g03126*	*Vitvi13g00716*	*Vitvi07g02538*	*Vitvi14g00465*	*Vitvi12g02628*	*Vitvi13g01337*
*Vitvi13g01956*	*Vitvi14g01647*	*Vitvi15g00889*	*Vitvi19g01788*	*Vitvi18g02954*	*Vitvi19g00198*
*Vitvi02g00055*	*Vitvi19g02128*	*Vitvi16g01480*	*Vitvi10g00751*	*Vitvi13g02410*	*Vitvi07g01644*
*Vitvi15g00522*	*Vitvi14g01646*	*Vitvi09g01605*	*Vitvi02g01442*	*Vitvi01g02166*	*Vitvi08g02386*
*Vitvi13g02064*	*Vitvi03g01773*	*Vitvi06g01559*	*Vitvi12g02254*	*Vitvi18g03336*	*Vitvi04g00470*
*Vitvi03g00678*	*Vitvi04g00407*	*Vitvi18g01264*	*Vitvi17g01613*	*Vitvi16g01603*	*Vitvi01g01236*
*Vitvi05g01276*	*Vitvi14g01642*	*Vitvi06g00024*	*Vitvi07g01856*	*Vitvi07g02720*	*Vitvi07g02029*
*Vitvi14g02934*	*Vitvi14g00301*	*Vitvi11g00165*	*Vitvi12g00632*	*Vitvi10g02060*	*Vitvi02g00286*
*Vitvi01g02237*	*Vitvi13g01307*	*Vitvi03g01596*	*Vitvi13g01888*	*Vitvi17g00994*	*Vitvi02g01022*
*Vitvi06g00008*	*Vitvi03g01877*	*Vitvi17g01465*	*Vitvi19g01825*	*Vitvi18g02969*	*Vitvi18g00628*
*Vitvi18g03094*	*Vitvi05g02089*	*Vitvi18g00882*	*Vitvi01g01181*	*Vitvi02g00126*	*Vitvi18g02780*
*Vitvi10g01552*	*Vitvi02g01003*	*Vitvi18g02589*	*Vitvi18g02495*	*Vitvi12g02291*	*Vitvi10g00559*
*Vitvi18g03001*	*Vitvi04g00831*	*Vitvi16g01262*	*Vitvi11g00431*	*Vitvi07g02393*	*Vitvi03g01431*
*Vitvi18g03358*	*Vitvi16g01886*	*Vitvi05g01860*	*Vitvi10g01825*	*Vitvi09g01801*	*Vitvi18g00189*
*Vitvi19g00962*	*Vitvi16g00160*	*Vitvi10g01857*	*Vitvi00g01122*	*Vitvi14g02786*	*Vitvi16g00504*
*Vitvi09g01210*	*Vitvi03g00924*	*Vitvi18g02630*	*Vitvi13g01425*	*Vitvi18g02028*	*Vitvi07g01552*
*Vitvi13g01638*	*Vitvi13g02605*	*Vitvi17g00293*	*Vitvi06g00380*	*Vitvi10g00960*	*Vitvi14g00065*
*Vitvi06g01099*	*Vitvi16g00122*	*Vitvi08g00793*	*Vitvi09g01951*	*Vitvi12g02255*	*Vitvi14g02458*
*Vitvi01g01054*	*Vitvi05g00963*	*Vitvi19g00039*	*Vitvi10g02187*	*Vitvi08g02455*	*Vitvi04g01801*
*Vitvi05g01435*	*Vitvi09g01569*	*Vitvi13g02369*	*Vitvi02g00697*	*Vitvi19g02168*	*Vitvi03g01232*
*Vitvi04g02195*	*Vitvi11g01231*	*Vitvi18g00473*	*Vitvi16g01169*	*Vitvi05g01738*	*Vitvi18g00131*
*Vitvi14g00041*	*Vitvi10g02128*	*Vitvi03g01352*	*Vitvi06g01324*	*Vitvi03g01619*	*Vitvi04g00524*
*Vitvi16g00275*	*Vitvi07g01867*	*Vitvi16g01336*	*Vitvi11g00010*	*Vitvi10g01635*	*Vitvi02g00433*
*Vitvi13g01102*	*Vitvi18g00143*	*Vitvi03g01350*	*Vitvi01g02137*	*Vitvi10g01596*	*Vitvi18g00510*
*Vitvi08g01055*	*Vitvi16g00118*	*Vitvi03g01651*	*Vitvi03g01857*	*Vitvi18g03399*	*Vitvi12g02200*
*Vitvi19g01631*	*Vitvi09g01792*	*Vitvi18g02617*	*Vitvi03g00427*	*Vitvi14g02649*	*Vitvi12g00025*
*Vitvi11g01660*	*Vitvi01g00066*	*Vitvi06g00665*	*Vitvi11g00081*	*Vitvi07g02372*	*Vitvi03g00439*
*Vitvi12g02476*	*Vitvi12g01264*	*Vitvi18g00260*	*Vitvi12g00290*	*Vitvi15g01655*	*Vitvi08g00820*
*Vitvi02g01758*	*Vitvi15g00078*	*Vitvi03g01196*	*Vitvi03g01412*	*Vitvi18g01777*	*Vitvi04g02049*
*Vitvi15g01652*	*Vitvi12g01917*	*Vitvi13g00066*	*Vitvi03g01679*	*Vitvi08g02426*	*Vitvi05g00170*
*Vitvi14g02521*	*Vitvi18g00107*	*Vitvi04g01389*	*Vitvi10g01774*	*Vitvi15g00874*	*Vitvi17g00885*
*Vitvi14g02721*	*Vitvi13g01873*	*Vitvi03g00473*	*Vitvi07g02071*	*Vitvi18g03079*	*Vitvi03g00023*
*Vitvi12g00080*	*Vitvi06g01599*	*Vitvi16g00890*	*Vitvi13g01085*	*Vitvi14g02537*	*Vitvi13g02292*
*Vitvi07g01718*	*Vitvi14g02584*	*Vitvi14g01390*	*Vitvi09g00966*	*Vitvi12g01879*	*Vitvi07g02588*
*Vitvi14g02749*	*Vitvi03g00925*	*Vitvi18g01320*	*Vitvi08g01205*	*Vitvi10g01595*	*Vitvi03g01729*
*Vitvi14g02513*	*Vitvi12g00644*	*Vitvi08g00886*	*Vitvi18g00609*	*Vitvi10g01617*	*Vitvi14g00398*
*Vitvi13g01013*	*Vitvi12g02475*	*Vitvi10g00562*	*Vitvi10g02191*	*Vitvi15g00875*	*Vitvi01g00821*
*Vitvi12g00039*	*Vitvi09g00409*	*Vitvi10g02389*	*Vitvi09g01584*	*Vitvi11g01553*	*Vitvi12g02599*
*Vitvi02g00424*	*Vitvi07g00301*	*Vitvi18g00313*	*Vitvi09g01983*	*Vitvi11g01418*	*Vitvi03g00338*
*Vitvi12g00037*	*Vitvi18g03314*	*Vitvi14g00952*	*Vitvi07g02112*	*Vitvi10g01293*	*Vitvi10g00621*
*Vitvi17g01101*	*Vitvi12g02458*	*Vitvi04g00641*	*Vitvi18g02404*	*Vitvi11g01699*	*Vitvi12g01841*
*Vitvi19g02316*	*Vitvi12g02318*	*Vitvi08g01572*	*Vitvi07g00063*	*Vitvi11g01654*	*Vitvi07g00337*
*Vitvi12g01838*	*Vitvi04g01638*	*Vitvi18g01238*	*Vitvi04g00884*	*Vitvi01g02146*	*Vitvi13g00070*
*Vitvi04g02153*	*Vitvi08g02056*	*Vitvi03g01348*	*Vitvi12g02657*	*Vitvi07g02258*	*Vitvi05g00402*
*Vitvi05g01288*	*Vitvi10g02215*	*Vitvi15g01495*	*Vitvi18g00366*	*Vitvi13g02539*	*Vitvi18g00691*
*Vitvi16g01529*	*Vitvi09g01770*	*Vitvi02g01407*	*Vitvi15g01204*	*Vitvi09g01675*	*Vitvi10g00442*
*Vitvi00g00889*	*Vitvi07g00899*	*Vitvi18g02730*	*Vitvi00g01940*	*Vitvi03g01859*	*Vitvi01g00264*
*Vitvi04g02194*	*Vitvi01g00739*	*Vitvi09g01581*	*Vitvi01g01836*	*Vitvi09g00902*	*Vitvi17g01318*
*Vitvi09g01141*	*Vitvi13g01945*	*Vitvi18g00061*	*Vitvi13g01969*	*Vitvi05g01985*	*Vitvi12g02147*
*Vitvi06g00610*	*Vitvi03g00702*	*Vitvi02g00017*	*Vitvi01g00566*	*Vitvi02g01673*	*Vitvi04g01799*
*Vitvi18g02060*	*Vitvi09g01181*	*Vitvi13g01001*	*Vitvi06g00351*	*Vitvi10g02061*	*Vitvi14g01637*
*Vitvi13g00127*	*Vitvi07g02109*	*Vitvi10g01624*	*Vitvi15g00754*	*Vitvi04g02061*	*Vitvi07g00542*
*Vitvi18g01914*	*Vitvi18g02455*	*Vitvi03g00936*	*Vitvi12g01832*	*Vitvi19g02066*	*Vitvi18g00268*
*Vitvi17g01397*	*Vitvi10g02170*	*Vitvi11g00365*	*Vitvi16g01779*	*Vitvi18g01585*	*Vitvi02g00052*
*Vitvi12g02641*	*Vitvi15g01687*	*Vitvi02g01705*	*Vitvi10g01795*	*Vitvi10g01993*	*Vitvi14g01341*
*Vitvi19g00484*	*Vitvi08g01293*	*Vitvi17g00955*	*Vitvi14g02455*	*Vitvi15g01448*	*Vitvi10g00399*
*Vitvi11g01105*	*Vitvi18g02628*	*Vitvi03g01358*	*Vitvi01g00680*	*Vitvi12g02284*	*Vitvi13g00143*
*Vitvi19g00412*	*Vitvi14g02408*	*Vitvi15g00641*	*Vitvi14g03087*	*Vitvi15g01722*	*Vitvi02g01446*
*Vitvi00g02220*	*Vitvi13g01874*	*Vitvi11g00838*	*Vitvi10g02081*	*Vitvi14g02737*	*Vitvi05g00377*
*Vitvi01g00796*	*Vitvi00g01102*	*Vitvi08g01348*	*Vitvi07g01658*	*Vitvi15g01379*	*Vitvi19g02064*
*Vitvi05g01903*	*Vitvi16g01884*	*Vitvi06g01921*	*Vitvi16g00879*	*Vitvi02g01590*	*Vitvi05g01901*
*Vitvi04g02197*	*Vitvi07g03001*	*Vitvi04g01635*	*Vitvi06g01638*	*Vitvi09g01918*	*Vitvi04g01653*
*Vitvi10g00535*	*Vitvi09g01791*	*Vitvi01g00864*	*Vitvi13g01861*	*Vitvi09g01820*	*Vitvi19g00682*
*Vitvi12g00002*	*Vitvi07g02477*	*Vitvi04g00735*	*Vitvi18g01168*	*Vitvi18g03021*	*Vitvi06g01459*
*Vitvi18g02857*	*Vitvi01g02000*	*Vitvi10g01715*	*Vitvi05g00126*	*Vitvi19g01041*	*Vitvi10g01756*
*Vitvi14g01402*	*Vitvi12g02179*	*Vitvi17g01520*	*Vitvi18g02661*	*Vitvi09g00918*	*Vitvi05g02177*
*Vitvi18g01376*	*Vitvi14g00775*	*Vitvi08g02121*	*Vitvi18g02648*	*Vitvi18g03389*	*Vitvi13g02075*
*Vitvi12g01911*	*Vitvi06g01944*	*Vitvi14g02558*	*Vitvi07g01759*	*Vitvi15g01749*	*Vitvi03g00397*
*Vitvi16g00022*	*Vitvi14g01645*	*Vitvi10g01029*	*Vitvi08g02381*	*Vitvi09g00808*	*Vitvi05g00864*
*Vitvi05g01469*	*Vitvi07g02368*	*Vitvi12g00251*	*Vitvi04g00801*	*Vitvi14g02675*	*Vitvi15g00396*
*Vitvi10g01651*	*Vitvi05g00604*	*Vitvi08g01342*	*Vitvi05g01259*	*Vitvi04g02060*	*Vitvi07g00176*
*Vitvi12g02125*	*Vitvi16g00129*	*Vitvi16g01175*	*Vitvi19g01727*	*Vitvi10g01823*	*Vitvi03g00443*
*Vitvi09g01371*	*Vitvi05g00381*	*Vitvi15g00921*	*Vitvi05g00861*	*Vitvi02g01784*	*Vitvi15g01021*
*Vitvi05g00523*	*Vitvi03g01531*	*Vitvi19g00041*	*Vitvi12g00148*	*Vitvi09g00884*	*Vitvi17g00098*
*Vitvi10g00519*	*Vitvi17g01395*	*Vitvi16g01979*	*Vitvi05g01828*	*Vitvi11g01360*	*Vitvi15g00915*
*Vitvi13g02425*	*Vitvi03g01525*	*Vitvi19g00397*	*Vitvi15g00929*	*Vitvi18g03317*	*Vitvi02g01358*
*Vitvi19g01061*	*Vitvi14g01805*	*Vitvi13g02005*	*Vitvi14g00243*	*Vitvi17g00593*	*Vitvi13g00076*
*Vitvi05g02199*	*Vitvi12g02639*	*Vitvi01g00477*	*Vitvi09g00241*	*Vitvi15g01717*	*Vitvi04g00241*
*Vitvi04g01895*	*Vitvi13g01684*	*Vitvi09g01398*	*Vitvi07g02228*	*Vitvi18g03345*	*Vitvi09g01409*
*Vitvi01g01886*	*Vitvi02g00521*	*Vitvi13g02009*	*Vitvi13g01871*	*Vitvi09g01956*	*Vitvi14g02990*
*Vitvi01g02020*	*Vitvi11g00634*	*Vitvi11g01681*	*Vitvi19g02118*	*Vitvi14g02545*	*Vitvi04g02087*
*Vitvi01g00815*	*Vitvi03g00411*	*Vitvi15g00537*	*Vitvi13g02023*	*Vitvi14g00220*	*Vitvi11g01532*
*Vitvi03g01571*	*Vitvi11g01634*	*Vitvi03g01597*	*Vitvi01g01437*	*Vitvi00g02009*	*Vitvi05g00046*
*Vitvi18g03182*	*Vitvi16g01123*	*Vitvi05g00360*	*Vitvi08g02368*	*Vitvi18g01839*	*Vitvi11g00727*
*Vitvi12g02259*	*Vitvi18g02993*	*Vitvi08g01789*	*Vitvi08g02325*	*Vitvi18g02942*	*Vitvi11g01428*
*Vitvi05g01453*	*Vitvi18g00204*	*Vitvi07g01734*	*Vitvi18g01637*	*Vitvi15g01331*	*Vitvi09g00995*
*Vitvi17g01476*	*Vitvi19g00479*	*Vitvi03g01361*	*Vitvi04g02308*	*Vitvi16g01651*	*Vitvi18g03254*
*Vitvi14g02522*	*Vitvi03g01522*	*Vitvi18g02075*	*Vitvi16g01952*	*Vitvi14g02873*	*Vitvi03g01430*
*Vitvi11g01471*	*Vitvi16g01800*	*Vitvi13g01744*	*Vitvi08g00108*	*Vitvi15g01343*	*Vitvi13g00405*
*Vitvi02g00811*	*Vitvi13g01876*	*Vitvi18g02616*	*Vitvi13g02043*	*Vitvi11g01497*	*Vitvi03g01428*
*Vitvi12g02231*	*Vitvi03g01742*	*Vitvi07g01847*	*Vitvi01g00892*	*Vitvi00g01834*	*Vitvi04g01795*
*Vitvi19g00052*	*Vitvi11g00950*	*Vitvi02g00022*	*Vitvi15g01647*	*Vitvi15g01739*	*Vitvi08g01089*
*Vitvi19g01863*	*Vitvi03g00699*	*Vitvi08g02126*	*Vitvi10g02083*	*Vitvi18g02701*	*Vitvi08g01276*
*Vitvi18g00623*	*Vitvi18g00816*	*Vitvi05g00478*	*Vitvi09g00021*	*Vitvi18g03024*	*Vitvi18g02754*
*Vitvi19g02236*	*Vitvi14g01318*	*Vitvi12g02434*	*Vitvi09g01603*	*Vitvi09g00916*	*Vitvi18g02399*
*Vitvi18g01763*	*Vitvi12g02213*	*Vitvi04g00060*	*Vitvi11g01266*	*Vitvi06g01870*	*Vitvi08g01602*
*Vitvi07g02711*	*Vitvi12g01172*	*Vitvi10g01628*	*Vitvi01g01885*	*Vitvi18g03350*	*Vitvi14g01865*
*Vitvi05g01156*	*Vitvi13g02592*	*Vitvi04g02239*	*Vitvi14g02477*	*Vitvi13g01254*	*Vitvi07g02433*
*Vitvi15g01425*	*Vitvi10g01699*	*Vitvi04g01160*	*Vitvi10g01017*	*Vitvi17g00009*	*Vitvi16g01352*
*Vitvi06g00547*	*Vitvi14g01907*	*Vitvi01g00612*	*Vitvi10g02214*	*Vitvi07g02673*	*Vitvi01g01654*
*Vitvi13g02459*	*Vitvi07g02413*	*Vitvi10g01023*	*Vitvi17g01427*	*Vitvi09g02096*	*Vitvi04g00527*
*Vitvi03g00574*	*Vitvi07g01837*	*Vitvi01g01808*	*Vitvi04g01264*	*Vitvi10g01392*	*Vitvi17g00565*
*Vitvi17g00224*	*Vitvi12g02523*	*Vitvi01g00656*	*Vitvi09g00717*	*Vitvi03g01737*	*Vitvi11g00183*
*Vitvi10g01930*	*Vitvi03g01819*	*Vitvi03g01364*	*Vitvi09g00472*	*Vitvi03g01674*	*Vitvi01g00424*
*Vitvi06g00672*	*Vitvi03g01530*	*Vitvi10g01623*	*Vitvi09g01490*	*Vitvi09g01526*	*Vitvi07g00807*
*Vitvi05g02208*	*Vitvi10g01455*	*Vitvi08g00868*	*Vitvi09g00621*	*Vitvi12g02762*	*Vitvi14g02811*
*Vitvi16g00715*	*Vitvi12g02317*	*Vitvi08g01024*	*Vitvi06g01574*	*Vitvi17g01539*	*Vitvi00g01020*
*Vitvi19g02392*	*Vitvi19g01636*	*Vitvi01g00608*	*Vitvi08g02282*	*Vitvi16g02154*	*Vitvi09g00007*
*Vitvi08g01846*	*Vitvi03g01528*	*Vitvi03g00144*	*Vitvi15g01641*	*Vitvi18g02899*	*Vitvi10g00276*
*Vitvi00g01239*	*Vitvi11g01442*	*Vitvi15g01035*	*Vitvi17g00556*	*Vitvi12g02257*	*Vitvi01g01038*
*Vitvi19g01371*	*Vitvi12g02324*	*Vitvi17g00292*	*Vitvi11g01366*	*Vitvi18g02901*	*Vitvi11g00317*
*Vitvi05g00109*	*Vitvi19g01525*	*Vitvi18g01098*	*Vitvi09g01851*	*Vitvi10g02044*	*Vitvi04g00106*
*Vitvi18g01919*	*Vitvi12g01431*	*Vitvi07g03142*	*Vitvi07g01728*	*Vitvi12g02605*	*Vitvi12g02495*
*Vitvi16g00900*	*Vitvi18g01849*	*Vitvi19g00141*	*Vitvi18g00746*	*Vitvi18g03025*	*Vitvi05g00486*
*Vitvi18g00153*	*Vitvi11g01437*	*Vitvi19g02057*	*Vitvi14g02599*	*Vitvi03g01785*	*Vitvi15g00906*
*Vitvi00g01276*	*Vitvi05g01944*	*Vitvi18g00274*	*Vitvi18g02534*	*Vitvi09g00886*	*Vitvi00g01061*
*Vitvi10g01910*	*Vitvi07g02404*	*Vitvi07g01737*	*Vitvi16g01132*	*Vitvi15g01356*	*Vitvi05g01786*
*Vitvi03g01591*	*Vitvi12g02634*	*Vitvi07g03112*	*Vitvi05g02255*	*Vitvi03g00933*	*Vitvi19g00153*
*Vitvi05g01419*	*Vitvi03g01535*	*Vitvi16g01176*	*Vitvi13g02139*	*Vitvi14g01946*	*Vitvi18g00435*
*Vitvi18g03095*	*Vitvi04g02252*	*Vitvi17g00046*	*Vitvi07g02858*	*Vitvi09g02095*	*Vitvi07g02265*
*Vitvi09g01914*	*Vitvi12g01323*	*Vitvi09g00193*	*Vitvi17g01357*	*Vitvi18g02896*	*Vitvi08g00014*
*Vitvi19g01832*	*Vitvi05g02253*	*Vitvi17g00977*	*Vitvi10g02241*	*Vitvi06g00266*	*Vitvi07g02240*
*Vitvi08g01972*	*Vitvi08g01235*	*Vitvi04g00680*	*Vitvi09g01982*	*Vitvi13g02143*	*Vitvi08g00030*
*Vitvi12g02701*	*Vitvi03g01482*	*Vitvi07g01773*	*Vitvi18g02838*	*Vitvi18g03083*	*Vitvi04g00636*
*Vitvi02g01355*	*Vitvi08g02266*	*Vitvi13g00669*	*Vitvi19g01896*	*Vitvi18g02902*	*Vitvi12g02138*
*Vitvi05g01617*	*Vitvi11g00001*	*Vitvi11g00811*	*Vitvi16g00971*	*Vitvi08g02464*	*Vitvi05g01571*
*Vitvi09g02007*	*Vitvi05g01945*	*Vitvi10g00605*	*Vitvi02g01325*	*Vitvi15g01389*	*Vitvi16g01164*
*Vitvi09g01790*	*Vitvi11g01576*	*Vitvi08g00970*	*Vitvi00g00989*	*Vitvi10g02105*	*Vitvi13g00591*
*Vitvi09g01912*	*Vitvi16g00787*	*Vitvi13g01932*	*Vitvi04g00696*	*Vitvi07g02373*	*Vitvi05g01350*
*Vitvi16g01699*	*Vitvi18g00878*	*Vitvi16g01418*	*Vitvi18g02591*	*Vitvi10g01195*	*Vitvi01g00673*
*Vitvi18g01959*	*Vitvi04g00432*	*Vitvi05g01269*	*Vitvi04g00136*	*Vitvi11g01357*	*Vitvi08g02163*
*Vitvi18g01877*	*Vitvi03g01486*	*Vitvi03g01363*	*Vitvi14g02655*	*Vitvi15g01665*	*Vitvi19g01902*
*Vitvi02g01354*	*Vitvi16g00349*	*Vitvi03g01292*	*Vitvi19g01948*	*Vitvi18g03038*	*Vitvi19g00515*
*Vitvi12g00452*	*Vitvi08g00267*	*Vitvi13g00012*	*Vitvi08g00155*	*Vitvi18g02949*	*Vitvi04g01095*
*Vitvi03g01540*	*Vitvi18g00444*	*Vitvi02g00327*	*Vitvi13g01267*	*Vitvi07g02378*	*Vitvi01g01304*
*Vitvi15g01705*	*Vitvi06g01060*	*Vitvi04g00289*	*Vitvi18g00807*	*Vitvi14g00223*	*Vitvi08g01767*
*Vitvi11g00882*	*Vitvi19g02009*	*Vitvi04g02238*	*Vitvi02g01744*	*Vitvi04g02018*	*Vitvi01g02158*
*Vitvi18g02198*	*Vitvi04g02310*	*Vitvi07g02990*	*Vitvi08g01503*	*Vitvi00g02357*	*Vitvi13g01107*
*Vitvi19g02286*	*Vitvi05g01200*	*Vitvi03g01872*	*Vitvi16g01325*	*Vitvi15g01323*	*Vitvi14g01344*
*Vitvi05g02141*	*Vitvi15g00675*	*Vitvi05g00067*	*Vitvi19g01601*	*Vitvi10g01519*	*Vitvi09g00599*
*Vitvi19g01217*		*Vitvi18g00687*	*Vitvi15g01642*	*Vitvi19g01821*	*Vitvi07g01454*
*Vitvi14g01938*		*Vitvi15g00753*	*Vitvi03g00047*	*Vitvi15g00494*	*Vitvi04g02313*
*Vitvi05g00485*		*Vitvi05g00802*	*Vitvi13g01245*	*Vitvi18g02905*	*Vitvi09g00223*
*Vitvi10g01431*		*Vitvi04g01934*	*Vitvi10g00439*	*Vitvi01g02168*	*Vitvi17g01050*
*Vitvi14g00025*		*Vitvi17g01389*	*Vitvi02g01363*	*Vitvi05g02073*	*Vitvi01g00292*
*Vitvi12g02153*		*Vitvi04g00722*	*Vitvi14g00401*	*Vitvi18g03341*	*Vitvi04g01097*
*Vitvi17g00306*		*Vitvi08g01190*	*Vitvi04g02221*	*Vitvi05g02103*	*Vitvi14g00185*
*Vitvi06g01765*		*Vitvi07g02987*	*Vitvi05g02050*	*Vitvi14g02536*	*Vitvi10g00946*
*Vitvi06g01334*		*Vitvi07g01240*	*Vitvi19g02049*	*Vitvi18g02936*	*Vitvi19g00164*
*Vitvi07g01714*		*Vitvi01g00638*	*Vitvi09g01980*	*Vitvi18g02947*	*Vitvi12g00026*
*Vitvi11g00013*		*Vitvi05g00637*	*Vitvi19g02025*	*Vitvi10g01989*	*Vitvi13g00278*
*Vitvi10g01941*		*Vitvi16g01078*	*Vitvi01g02124*	*Vitvi10g01201*	*Vitvi02g00938*
*Vitvi18g03362*		*Vitvi02g00532*	*Vitvi14g02868*	*Vitvi15g01677*	*Vitvi07g01596*
*Vitvi05g02085*		*Vitvi13g00375*	*Vitvi14g02781*	*Vitvi18g02953*	*Vitvi02g00374*
*Vitvi14g00577*		*Vitvi14g03042*	*Vitvi09g01742*	*Vitvi16g01764*	*Vitvi08g00808*
*Vitvi14g02692*		*Vitvi10g02177*	*Vitvi12g02737*	*Vitvi18g02944*	*Vitvi08g01126*
*Vitvi05g01153*		*Vitvi06g01830*	*Vitvi14g02497*	*Vitvi04g00352*	*Vitvi02g00223*
*Vitvi14g00707*		*Vitvi07g00400*	*Vitvi06g01711*	*Vitvi15g00323*	*Vitvi03g00378*
*Vitvi18g01962*		*Vitvi18g01735*	*Vitvi14g03007*	*Vitvi16g01622*	*Vitvi19g00029*
*Vitvi04g00433*		*Vitvi19g00735*	*Vitvi06g00359*	*Vitvi16g01738*	*Vitvi09g00582*
*Vitvi17g00309*		*Vitvi16g00071*	*Vitvi14g00997*	*Vitvi16g00859*	*Vitvi17g01593*
*Vitvi12g01861*		*Vitvi09g01122*	*Vitvi07g00822*	*Vitvi07g00478*	*Vitvi02g00228*
*Vitvi01g00088*		*Vitvi12g00721*	*Vitvi12g02258*	*Vitvi09g01142*	*Vitvi07g01354*
*Vitvi16g01278*		*Vitvi07g02989*	*Vitvi15g01560*	*Vitvi18g03061*	*Vitvi11g01602*
*Vitvi13g02470*		*Vitvi09g00185*	*Vitvi16g01312*	*Vitvi19g01083*	*Vitvi14g02009*
*Vitvi12g01908*		*Vitvi09g01954*	*Vitvi04g02207*	*Vitvi13g02439*	*Vitvi06g00329*
*Vitvi19g01820*		*Vitvi18g01253*	*Vitvi05g01611*	*Vitvi10g01829*	*Vitvi10g00209*
*Vitvi03g00504*		*Vitvi07g02573*	*Vitvi13g02489*	*Vitvi09g00682*	*Vitvi06g00785*
*Vitvi16g02028*		*Vitvi11g00360*	*Vitvi13g00388*	*Vitvi02g01280*	*Vitvi07g02634*
*Vitvi05g02155*		*Vitvi11g00417*	*Vitvi12g00648*	*Vitvi16g01760*	*Vitvi01g00309*
*Vitvi13g00940*		*Vitvi11g00089*	*Vitvi05g01451*	*Vitvi18g03067*	*Vitvi10g01641*
*Vitvi18g01716*			*Vitvi08g01991*	*Vitvi00g02238*	*Vitvi02g00265*
*Vitvi05g01923*			*Vitvi05g01768*	*Vitvi19g01836*	*Vitvi18g00149*
*Vitvi13g02026*			*Vitvi05g02093*	*Vitvi14g02554*	*Vitvi03g01337*
*Vitvi18g02649*			*Vitvi05g01370*	*Vitvi16g00391*	*Vitvi07g01078*
*Vitvi08g01110*			*Vitvi10g02035*	*Vitvi17g01046*	*Vitvi07g03014*
*Vitvi07g01045*			*Vitvi08g01938*	*Vitvi00g01769*	*Vitvi13g01066*
*Vitvi17g01286*			*Vitvi05g00411*	*Vitvi18g03022*	*Vitvi04g00846*
*Vitvi07g02077*			*Vitvi18g02656*	*Vitvi19g01929*	*Vitvi15g01575*
*Vitvi19g01850*			*Vitvi03g00953*	*Vitvi02g01764*	*Vitvi09g01204*
*Vitvi04g01836*			*Vitvi14g00296*	*Vitvi05g02036*	*Vitvi06g00709*
*Vitvi19g01824*			*Vitvi05g02147*	*Vitvi15g01330*	*Vitvi02g00409*
*Vitvi11g01181*			*Vitvi12g00549*	*Vitvi17g01673*	*Vitvi08g01470*
*Vitvi05g00905*			*Vitvi05g02144*	*Vitvi18g02703*	*Vitvi07g02595*
*Vitvi18g03217*			*Vitvi09g00475*	*Vitvi05g00152*	*Vitvi12g00414*
*Vitvi18g00154*			*Vitvi06g00078*	*Vitvi10g01620*	*Vitvi01g00246*
*Vitvi13g02473*			*Vitvi05g01530*	*Vitvi15g00918*	*Vitvi09g02017*
*Vitvi07g01719*			*Vitvi15g01450*	*Vitvi09g00914*	*Vitvi17g01122*
*Vitvi09g00356*			*Vitvi13g00948*	*Vitvi19g01950*	*Vitvi13g01556*
*Vitvi09g00867*			*Vitvi10g01121*	*Vitvi12g02763*	*Vitvi02g00719*
*Vitvi01g00873*			*Vitvi04g02214*	*Vitvi12g02189*	*Vitvi19g00071*
*Vitvi13g00037*			*Vitvi13g00458*	*Vitvi18g02698*	*Vitvi13g01778*
*Vitvi01g01949*			*Vitvi10g00478*	*Vitvi13g01676*	*Vitvi08g02168*
*Vitvi18g00470*			*Vitvi16g00515*	*Vitvi16g01461*	*Vitvi11g00022*
*Vitvi12g02726*			*Vitvi14g01053*	*Vitvi07g02665*	*Vitvi08g02184*
*Vitvi14g02004*			*Vitvi06g01233*	*Vitvi12g02730*	*Vitvi07g01329*
*Vitvi18g00191*			*Vitvi03g01336*	*Vitvi13g02423*	*Vitvi11g00336*
*Vitvi13g02028*			*Vitvi18g00604*	*Vitvi08g02081*	*Vitvi01g00637*
*Vitvi13g00425*			*Vitvi02g00181*	*Vitvi18g02873*	*Vitvi07g01816*
*Vitvi15g01710*			*Vitvi08g02221*	*Vitvi01g02134*	*Vitvi01g02263*
*Vitvi19g00101*			*Vitvi13g02241*	*Vitvi09g00809*	*Vitvi07g01273*
*Vitvi11g01270*			*Vitvi14g02977*	*Vitvi18g03065*	*Vitvi12g00560*
*Vitvi16g01701*			*Vitvi16g01491*	*Vitvi10g01599*	*Vitvi04g01261*
*Vitvi00g02329*			*Vitvi01g02178*	*Vitvi19g02229*	*Vitvi17g01625*
*Vitvi05g00930*			*Vitvi15g00570*	*Vitvi16g01465*	*Vitvi07g02969*
*Vitvi01g02105*			*Vitvi01g00546*	*Vitvi13g02583*	*Vitvi18g00181*
*Vitvi13g00474*			*Vitvi04g00774*	*Vitvi16g00321*	*Vitvi10g00687*
			*Vitvi16g01955*	*Vitvi10g01598*	*Vitvi10g01605*
			*Vitvi03g01098*	*Vitvi18g03356*	*Vitvi16g01103*
			*Vitvi08g01369*	*Vitvi08g02159*	*Vitvi04g00264*
			*Vitvi18g01533*	*Vitvi12g01701*	*Vitvi15g00998*
			*Vitvi17g01373*	*Vitvi18g03027*	*Vitvi12g00221*
			*Vitvi09g01553*	*Vitvi18g03351*	*Vitvi06g00494*
			*Vitvi08g02046*	*Vitvi15g01253*	*Vitvi12g00086*
			*Vitvi10g01095*	*Vitvi03g01682*	*Vitvi01g02030*
				*Vitvi15g00308*	*Vitvi12g00542*
				*Vitvi03g00407*	*Vitvi14g01685*
				*Vitvi16g01824*	*Vitvi09g00568*
				*Vitvi07g02360*	*Vitvi06g00518*
				*Vitvi05g02210*	*Vitvi08g01724*
				*Vitvi14g02552*	*Vitvi05g00539*
				*Vitvi15g01704*	*Vitvi05g02190*
				*Vitvi17g01464*	*Vitvi06g01057*
				*Vitvi07g02357*	*Vitvi08g02261*
				*Vitvi07g02315*	*Vitvi05g00736*
				*Vitvi18g03039*	*Vitvi07g00015*
				*Vitvi03g01732*	*Vitvi04g02297*
				*Vitvi14g02532*	*Vitvi10g01540*
				*Vitvi04g00723*	*Vitvi02g01778*
				*Vitvi08g02255*	*Vitvi05g01833*
				*Vitvi16g01459*	*Vitvi08g02225*
				*Vitvi18g02668*	*Vitvi17g01474*
				*Vitvi08g00728*	*Vitvi05g01855*
				*Vitvi16g02161*	*Vitvi08g01011*
				*Vitvi10g01313*	*Vitvi18g00283*
				*Vitvi04g02215*	*Vitvi04g00047*
				*Vitvi15g01419*	*Vitvi16g01830*
				*Vitvi18g03033*	*Vitvi08g01406*
				*Vitvi01g02143*	*Vitvi10g01103*
				*Vitvi02g00809*	*Vitvi05g01113*
				*Vitvi14g02534*	*Vitvi09g01273*
				*Vitvi18g02895*	*Vitvi19g00113*
				*Vitvi12g02425*	*Vitvi04g02296*
				*Vitvi09g01957*	*Vitvi06g00074*
				*Vitvi03g01446*	*Vitvi06g00202*
				*Vitvi09g01778*	*Vitvi08g01839*
				*Vitvi01g02311*	*Vitvi10g00612*
				*Vitvi18g02723*	*Vitvi13g01068*
				*Vitvi12g02662*	*Vitvi07g01766*
				*Vitvi10g01824*	*Vitvi19g01917*
				*Vitvi17g00458*	*Vitvi06g01709*
				*Vitvi14g02787*	*Vitvi10g00711*
				*Vitvi10g02008*	*Vitvi05g00128*
				*Vitvi09g02051*	*Vitvi14g01721*
				*Vitvi10g00504*	*Vitvi18g00694*
				*Vitvi03g01359*	*Vitvi09g01519*
				*Vitvi09g01757*	*Vitvi05g00337*
				*Vitvi15g01360*	*Vitvi05g00218*
				*Vitvi07g00957*	*Vitvi07g01363*
				*Vitvi05g01982*	*Vitvi16g00553*
				*Vitvi17g01630*	*Vitvi10g00465*
				*Vitvi06g01720*	*Vitvi17g00892*
				*Vitvi04g02193*	*Vitvi04g00252*
				*Vitvi18g03174*	*Vitvi14g02461*
				*Vitvi03g01640*	*Vitvi10g00694*
				*Vitvi10g02052*	*Vitvi10g01250*
				*Vitvi03g01663*	*Vitvi04g01296*
				*Vitvi16g01608*	*Vitvi17g00757*
				*Vitvi09g01971*	*Vitvi14g01305*
				*Vitvi15g01319*	*Vitvi16g01217*
				*Vitvi14g00676*	*Vitvi01g00363*
				*Vitvi11g00982*	*Vitvi08g01040*
				*Vitvi12g01849*	*Vitvi16g02078*
				*Vitvi04g00349*	*Vitvi07g01325*
				*Vitvi12g02182*	*Vitvi18g02813*
				*Vitvi18g03403*	*Vitvi10g00880*
				*Vitvi00g01835*	*Vitvi11g00284*
				*Vitvi18g02104*	*Vitvi07g00444*
				*Vitvi04g00874*	*Vitvi04g00090*
				*Vitvi00g01893*	*Vitvi10g00266*
				*Vitvi19g02363*	*Vitvi02g01644*
				*Vitvi12g00509*	*Vitvi04g01802*
				*Vitvi09g01626*	*Vitvi00g00894*
				*Vitvi12g01154*	*Vitvi08g01550*
				*Vitvi10g01964*	*Vitvi06g00548*
				*Vitvi14g03088*	*Vitvi14g02462*
				*Vitvi16g00442*	*Vitvi13g00994*
				*Vitvi03g01714*	*Vitvi16g00518*
				*Vitvi09g02085*	*Vitvi09g00355*
				*Vitvi01g02312*	*Vitvi08g01013*
				*Vitvi12g00920*	*Vitvi19g00285*
				*Vitvi01g02156*	*Vitvi10g00705*
				*Vitvi03g01793*	*Vitvi17g01275*
					*Vitvi06g00465*
					*Vitvi06g00569*
					*Vitvi18g01217*
					*Vitvi06g00299*
					*Vitvi14g00902*
					*Vitvi14g02457*
					*Vitvi07g02753*
					*Vitvi07g00105*
					*Vitvi14g00152*
					*Vitvi14g01859*
					*Vitvi06g01635*
					*Vitvi00g00936*
					*Vitvi17g00918*
					*Vitvi14g01524*
					*Vitvi03g00579*
					*Vitvi17g00384*
					*Vitvi16g01126*
					*Vitvi01g01583*
					*Vitvi06g00774*
					*Vitvi16g00921*
					*Vitvi13g00865*
					*Vitvi19g02276*
					*Vitvi02g00499*
					*Vitvi17g00030*
					*Vitvi09g00187*
					*Vitvi15g01146*
					*Vitvi05g00684*
					*Vitvi18g00708*
					*Vitvi18g02420*
					*Vitvi02g01314*
					*Vitvi12g00771*
					*Vitvi04g01664*
					*Vitvi11g00231*
					*Vitvi01g00941*
					*Vitvi06g00169*
					*Vitvi04g01100*
					*Vitvi11g00413*
					*Vitvi09g00647*
					*Vitvi05g00841*
					*Vitvi06g00757*
					*Vitvi03g00104*
					*Vitvi09g00166*
					*Vitvi04g01883*
					*Vitvi05g01848*
					*Vitvi03g01328*
					*Vitvi12g00729*
					*Vitvi10g00011*
					*Vitvi19g02045*
					*Vitvi11g00459*
					*Vitvi13g00270*
					*Vitvi05g02077*
					*Vitvi15g00989*
					*Vitvi14g00063*
					*Vitvi06g01621*
					*Vitvi17g00631*
					*Vitvi05g02181*
					*Vitvi08g00145*
					*Vitvi10g01086*
					*Vitvi02g00025*
					*Vitvi04g01529*
					*Vitvi18g01170*
					*Vitvi01g00078*
					*Vitvi00g01106*
					*Vitvi01g00648*
					*Vitvi18g01237*
					*Vitvi19g00572*
					*Vitvi19g00190*
					*Vitvi12g00383*
					*Vitvi14g01796*
					*Vitvi06g01673*
					*Vitvi10g00470*
					*Vitvi14g01894*
					*Vitvi07g01333*
					*Vitvi16g00024*
					*Vitvi04g00091*
					*Vitvi02g00482*
					*Vitvi18g00145*
					*Vitvi03g00230*
					*Vitvi19g00470*
					*Vitvi05g00575*
					*Vitvi09g00772*
					*Vitvi17g00578*
					*Vitvi01g00825*
					*Vitvi14g01984*
					*Vitvi19g00012*
					*Vitvi01g00150*
					*Vitvi01g00903*
					*Vitvi18g01173*
					*Vitvi05g00513*
					*Vitvi02g01274*
					*Vitvi07g00457*
					*Vitvi18g02501*
					*Vitvi07g02191*
					*Vitvi05g00770*
					*Vitvi02g01779*
					*Vitvi05g00535*
					*Vitvi03g01432*
					*Vitvi05g00628*
					*Vitvi15g00740*
					*Vitvi05g01048*
					*Vitvi07g02172*
					*Vitvi08g01119*
					*Vitvi19g00182*
					*Vitvi07g02971*
					*Vitvi08g00217*
					*Vitvi01g01826*
					*Vitvi10g02169*
					*Vitvi11g01241*
					*Vitvi15g00691*
					*Vitvi16g01135*
					*Vitvi05g00538*
					*Vitvi13g01253*
					*Vitvi13g02017*
					*Vitvi09g00045*
					*Vitvi15g00886*
					*Vitvi12g00714*
					*Vitvi18g00055*
					*Vitvi12g00426*
					*Vitvi02g01192*
					*Vitvi04g01159*
					*Vitvi08g01408*
					*Vitvi16g00183*
					*Vitvi18g01661*
					*Vitvi03g01805*
					*Vitvi00g00951*
					*Vitvi10g01471*
					*Vitvi02g00454*
					*Vitvi09g00165*
					*Vitvi04g00317*
					*Vitvi07g00701*
					*Vitvi08g00798*
					*Vitvi07g00048*
					*Vitvi05g00012*
					*Vitvi06g00944*
					*Vitvi13g00274*
					*Vitvi18g02052*
					*Vitvi04g02184*
					*Vitvi14g00475*
					*Vitvi14g02562*
					*Vitvi12g00486*
					*Vitvi10g00113*
					*Vitvi08g02411*
					*Vitvi18g00795*
					*Vitvi04g01792*
					*Vitvi17g00634*
					*Vitvi14g00495*
					*Vitvi01g01173*
					*Vitvi04g00210*
					*Vitvi02g01313*
					*Vitvi11g00411*
					*Vitvi10g02166*
					*Vitvi00g01333*
					*Vitvi07g02170*
					*Vitvi10g01360*
					*Vitvi17g00415*
					*Vitvi04g01800*
					*Vitvi18g02796*
					*Vitvi02g00018*
					*Vitvi00g00816*
					*Vitvi17g00071*
					*Vitvi11g00249*
					*Vitvi14g01730*
					*Vitvi05g01117*
					*Vitvi02g01211*
					*Vitvi17g00661*
					*Vitvi00g01309*
					*Vitvi02g00563*
					*Vitvi06g01119*
					*Vitvi06g01000*
					*Vitvi12g00144*
					*Vitvi18g00719*
					*Vitvi01g01476*
					*Vitvi17g01025*
					*Vitvi08g01841*
					*Vitvi16g01306*
					*Vitvi18g01088*
					*Vitvi06g00625*
					*Vitvi16g00184*
					*Vitvi13g02507*
					*Vitvi11g00278*
					*Vitvi09g00198*
					*Vitvi06g00943*
					*Vitvi10g01422*
					*Vitvi10g01075*
					*Vitvi14g00289*
					*Vitvi01g00296*
					*Vitvi06g00538*
					*Vitvi16g01014*
					*Vitvi08g02186*
					*Vitvi06g01113*
					*Vitvi07g03100*
					*Vitvi14g00396*
					*Vitvi04g00184*
					*Vitvi14g01877*
					*Vitvi19g00652*
					*Vitvi07g01410*
					*Vitvi12g02143*
					*Vitvi04g00397*
					*Vitvi03g00376*
					*Vitvi08g01835*
					*Vitvi14g02566*
					*Vitvi03g00717*
					*Vitvi18g01448*
					*Vitvi17g00171*
					*Vitvi01g01459*
					*Vitvi10g01318*
					*Vitvi04g00962*
					*Vitvi05g00325*
					*Vitvi10g01456*
					*Vitvi13g02024*
					*Vitvi07g00581*
					*Vitvi02g00194*
					*Vitvi13g01743*
					*Vitvi18g00888*
					*Vitvi12g00576*
					*Vitvi02g01005*
					*Vitvi03g00182*
					*Vitvi16g00928*
					*Vitvi17g00114*
					*Vitvi12g00755*
					*Vitvi14g01470*
					*Vitvi14g00233*
					*Vitvi09g00997*
					*Vitvi18g01172*
					*Vitvi08g01616*
					*Vitvi09g00629*
					*Vitvi06g00506*
					*Vitvi08g01891*
					*Vitvi14g01858*
					*Vitvi12g00681*
					*Vitvi10g00702*
					*Vitvi07g02242*
					*Vitvi08g01143*

## References

[1] Chaves M.M., Zarrouk O., Francisco R., Costa J.M., Santos T., Regalado A.P., Rodrigues M.L., Lopes C.M. (2010). Grapevine under deficit irrigation: Hints from physiological and molecular data. Ann. Bot..

[2] Pou A., Medrano H., Tomàs M., Martorell S., Ribas-Carbó M., Flexas J. (2012). Anisohydric behaviour in grapevines results in better performance under moderate water stress and recovery than isohydric behaviour. Plant Soil.

[3] Hochberg U., Degu A., Fait A., Rachmilevitch S. (2013). Near isohydric grapevine cultivar displays higher photosynthetic efficiency and photorespiration rates under drought stress as compared with near anisohydric grapevine cultivar. Physiol. Plant..

[4] Martínez-Vilalta J., Garcia-Forner N. (2017). Water potential regulation, stomatal behaviour and hydraulic transport under drought: Deconstructing the iso/anisohydric concept. Plant Cell Environ..

[5] Schultz H.R. (2003). Differences in hydraulic architecture account for near-isohydric and anisohydric behaviour of two field-grown *Vitis vinifera* L. cultivars during drought. Plant Cell Environ..

[6] Soar C.J., Speirs J., Maffei S.M., Penrose A.B., McCarthy M.G., Loveys B.R. (2006). Grape vine varieties Shiraz and Grenache differ in their stomatal response to VPD: Apparent links with ABA physiology and gene expression in leaf tissue. Aust. J. Grape Wine Res..

[7] Tombesi S., Nardini A., Farinelli D., Palliotti A. (2014). Relationships between stomatal behavior, xylem vulnerability to cavitation and leaf water relations in two cultivars of *Vitis vinifera*. Physiol. Plant..

[8] Lavoie-Lamoureux A., Sacco D., Risse P.-A., Lovisolo C. (2017). Factors influencing stomatal conductance in response to water availability in grapevine: A meta-analysis. Physiol. Plant..

[9] Serrano A.S., Martínez-Gascueña J., Chacón-Vozmediano J.L. (2024). Variability in water use behavior during drought of different grapevine varieties: Assessment of their regulation of water status and stomatal control. Agric. Water Manag..

[10] Rogiers S.Y., Greer D.H., Hatfield J.M., Hutton R.J., Clarke S.J., Hutchinson P.A., Somers A. (2012). Stomatal response of an anisohydric grapevine cultivar to evaporative demand, available soil moisture and abscisic acid. Tree Physiol..

[11] Garcia-Forner N., Biel C., Savé R., Martínez-Vilalta J. (2017). Isohydric species are not necessarily more carbon limited than anisohydric species during drought. Meinzer, F.; editor. Tree Physiol..

[12] Yi K., Dragoni D., Phillips R.P., Roman D.T., Novick K.A. (2017). Dynamics of stem water uptake among isohydric and anisohydric species experiencing a severe drought. Tree Physiol..

[13] McDowell N., Pockman W.T., Allen C.D., Breshears D.D., Cobb N., Kolb T., Plaut J., Sperry J., West A., Williams D.G. (2008). Mechanisms of plant survival and mortality during drought: Why do some plants survive while others succumb to drought?. New Phytol..

[14] Dayer S., Scharwies J.D., Ramesh S.A., Sullivan W., Doerflinger F.C., Pagay V., Tyerman S.D. (2020). Comparing Hydraulics Between Two Grapevine Cultivars Reveals Differences in Stomatal Regulation Under Water Stress and Exogenous ABA Applications. Front Plant Sci..

[15] Santo S.D., Palliotti A., Zenoni S., Tornielli G.B., Fasoli M., Paci P., Tombesi S., Frioni T., Silvestroni O., Bellincontro A. (2016). Distinct transcriptome responses to water limitation in isohydric and anisohydric grapevine cultivars. BMC Genom..

[16] Villalobos-González L., Muñoz-Araya M., Franck N., Pastenes C. (2019). Controversies in Midday Water Potential Regulation and Stomatal Behavior Might Result from the Environment, Genotype, and/or Rootstock: Evidence from Carménère and Syrah Grapevine Varieties. Front. Plant Sci..

[17] Fu X., Meinzer F.C. (2019). Metrics and proxies for stringency of regulation of plant water status (iso/anisohydry): A global data set reveals coordination and trade-offs among water transport traits. Tree Physiol..

[18] Konecny T., Nikoghosyan M., Binder H. (2023). Machine learning extracts marks of thiamine’s role in cold acclimation in the transcriptome of *Vitis vinifera*. Front. Plant Sci..

[19] Tusher V.G., Tibshirani R., Chu G. (2001). Significance analysis of microarrays applied to the ionizing radiation response. Proc. Natl. Acad. Sci. USA.

[20] Wirth H., Löffler M., Von Bergen M., Binder H. (2011). Expression cartography of human tissues using self organizing maps. BMC Bioinform..

[21] Yang X., Lu M., Wang Y., Wang Y., Liu Z., Chen S. (2021). Response Mechanism of Plants to Drought Stress. Horticulturae.

[22] Urao T., Yamaguchi-Shinozaki K., Urao S., Shinozaki K. (1993). An Arabidopsis myb homolog is induced by dehydration stress and its gene product binds to the conserved MYB recognition sequence. Plant Cell.

[23] Chen B.-J., Wang Y., Hu Y.-L., Wu Q., Lin Z.-P. (2005). Cloning and characterization of a drought-inducible MYB gene from *Boea crassifolia*. Plant Sci..

[24] Jung C., Seo J.S., Han S.W., Koo Y.J., Kim C.H., Song S.I., Nahm B.H., Choi Y.D., Cheong J.-J. (2008). Overexpression of AtMYB44 Enhances Stomatal Closure to Confer Abiotic Stress Tolerance in Transgenic Arabidopsis. Plant Physiol..

[25] SSeo P.J., Xiang F., Qiao M., Park J.-Y., Na Lee Y., Kim S.-G., Lee Y.-H., Park W.J., Park C.-M. (2009). The MYB96 Transcription Factor Mediates Abscisic Acid Signaling during Drought Stress Response in Arabidopsis. Plant Physiol..

[26] Prabu G., Prasad D.T. (2012). Functional characterization of sugarcane MYB transcription factor gene promoter (PScMYBAS1) in response to abiotic stresses and hormones. Plant Cell Rep..

[27] Wang X., Niu Y., Zheng Y. (2021). Multiple Functions of MYB Transcription Factors in Abiotic Stress Responses. Int. J. Mol. Sci..

[28] Li S., Khoso M.A., Wu J., Yu B., Wagan S., Liu L. (2024). Exploring the mechanisms of WRKY transcription factors and regulated pathways in response to abiotic stress. Plant Stress.

[29] Ando S., Nomoto M., Iwakawa H., Vial-Pradel S., Luo L., Sasabe M., Ohbayashi I., Yamamoto K.T., Tada Y., Sugiyama M. (2023). Arabidopsis ASYMMETRIC LEAVES2 and Nucleolar Factors Are Coordinately Involved in the Perinucleolar Patterning of AS2 Bodies and Leaf Development. Plants.

[30] Hyndman D., Bauman D.R., Heredia V.V., Penning T.M. (2003). The aldo-keto reductase superfamily homepage. Chem.-Biol. Interact..

[31] Kirankumar T.V., Madhusudhan K.V., Nareshkumar A., Kiranmai K., Lokesh U., Venkatesh B., Sudhakar C. (2016). Expression Analysis of Aldo-Keto Reductase 1 (AKR1) in Foxtail Millet (*Setaria italica* L.) Subjected to Abiotic Stresses. Am. J. Plant Sci..

[32] Boncan D.A.T., Tsang S.S., Li C., Lee I.H., Lam H.-M., Chan T.-F., Hui J.H. (2020). Terpenes and Terpenoids in Plants: Interactions with Environment and Insects. Int. J. Mol. Sci..

[33] Tu M., Wang X., Yin W., Wang Y., Li Y., Zhang G., Li Z., Song J., Wang X. (2020). Grapevine VlbZIP30 improves drought resistance by directly activating VvNAC17 and promoting lignin biosynthesis through the regulation of three peroxidase genes. Hortic. Res..

[34] Han X., Zhao Y., Chen Y., Xu J., Jiang C., Wang X., Zhuo R., Lu M.-Z., Zhang J. (2022). Lignin biosynthesis and accumulation in response to abiotic stresses in woody plants. For. Res..

[35] Malerba M., Cerana R. (2020). Chitin- and Chitosan-Based Derivatives in Plant Protection against Biotic and Abiotic Stresses and in Recovery of Contaminated Soil and Water. Polysaccharides.

[36] Pratyusha D.S., Sarada D.V.L. (2022). MYB transcription factors—Master regulators of phenylpropanoid biosynthesis and diverse developmental and stress responses. Plant Cell Rep..

[37] Czemmel S., Stracke R., Weisshaar B., Cordon N., Harris N.N., Walker A.R., Robinson S.P., Bogs J. (2009). The Grapevine R2R3-MYB Transcription Factor VvMYBF1 Regulates Flavonol Synthesis in Developing Grape Berries. Plant Physiol..

[38] Machida Y., Suzuki T., Sasabe M., Iwakawa H., Kojima S., Machida C. (2022). Arabidopsis ASYMMETRIC LEAVES2 (AS2): Roles in plant morphogenesis, cell division, and pathogenesis. J. Plant Res..

[39] Goyal P., Devi R., Verma B., Hussain S., Arora P., Tabassum R., Gupta S. (2023). WRKY transcription factors: Evolution, regulation, and functional diversity in plants. Protoplasma.

[40] Huang H., Ullah F., Zhou D.-X., Yi M., Zhao Y. (2019). Mechanisms of ROS Regulation of Plant Development and Stress Responses. Front. Plant Sci..

[41] Kumari S., Nazir F., Maheshwari C., Kaur H., Gupta R., Siddique K.H., Khan M.I.R. (2024). Plant hormones and secondary metabolites under environmental stresses: Enlightening defense molecules. Plant Physiol. Biochem..

[42] Muhammad D., Alameldin H.F., Oh S., Montgomery B.L., Warpeha K.M. (2023). Arogenate dehydratases: Unique roles in light-directed development during the seed-to-seedling transition in *Arabidopsis thaliana*. Front. Plant Sci..

[43] Singh M., Kumar J., Singh S., Singh V.P., Prasad S.M. (2015). Roles of osmoprotectants in improving salinity and drought tolerance in plants: A review. Rev. Environ. Sci. Bio. Technol..

[44] Xu Z., Zhang D., Hu J., Zhou X., Ye X., Reichel K.L., Stewart N.R., Syrenne R.D., Yang X., Gao P. (2009). Comparative genome analysis of lignin biosynthesis gene families across the plant kingdom. BMC Bioinform..

[45] Lu Y., Shao D., Shi J., Huang Q., Yang H., Jin M. (2016). Strategies for enhancing resveratrol production and the expression of pathway enzymes. Appl. Microbiol. Biotechnol..

[46] Dubrovina A.S., Kiselev K.V. (2017). Regulation of stilbene biosynthesis in plants. Planta.

[47] Roy S., Mishra M., Dhankher O.P., Singla-Pareek S.L., Pareek A., Rajpal V.R., Sehgal D., Kumar A., Raina S.N. (2019). Molecular Chaperones: Key Players of Abiotic Stress Response in Plants. Genetic Enhancement of Crops for Tolerance to Abiotic Stress: Mechanisms and Approaches, Vol I.

[48] Khatri P., Chen L., Rajcan I., Dhaubhadel S. (2023). Functional characterization of *Cinnamate 4-hydroxylase* gene family in soybean (*Glycine max*). PLoS ONE.

[49] Kriegshauser L., Knosp S., Grienenberger E., Tatsumi K., Gütle D.D., Sørensen I., Herrgott L., Zumsteg J., Rose J.K.C., Reski R. (2021). Function of the HYDROXYCINNAMOYL-CoA:SHIKIMATE HYDROXYCINNAMOYL TRANSFERASE is evolutionarily conserved in embryophytes. Plant Cell.

[50] Serrani-Yarce J.C., Escamilla-Trevino L., Barros J., Gallego-Giraldo L., Pu Y., Ragauskas A., Dixon R.A. (2021). Targeting hydroxycinnamoyl CoA: Shikimate hydroxycinnamoyl transferase for lignin modification in *Brachypodium distachyon*. Biotechnol. Biofuels.

[51] Petersen M. (2016). Hydroxycinnamoyltransferases in plant metabolism. Phytochem. Rev..

[52] Giordano D., Provenzano S., Ferrandino A., Vitali M., Pagliarani C., Roman F., Cardinale F., Castellarin S.D., Schubert A. (2016). Characterization of a multifunctional caffeoyl-CoA O-methyltransferase activated in grape berries upon drought stress. Plant Physiol. Biochem..

[53] Austin M.B., Bowman M.E., Ferrer J.-L., Schröder J., Noel J.P. (2004). An Aldol Switch Discovered in Stilbene Synthases Mediates Cyclization Specificity of Type III Polyketide Synthases. Chem. Biol..

[54] Schmidlin L., Poutaraud A., Claudel P., Mestre P., Prado E., Santos-Rosa M., Wiedemann-Merdinoglu S., Karst F., Merdinoglu D., Hugueney P. (2008). A Stress-Inducible Resveratrol O-Methyltransferase Involved in the Biosynthesis of Pterostilbene in Grapevine. Plant Physiol..

[55] Tunc-Ozdemir M., Miller G., Song L., Kim J., Sodek A., Koussevitzky S., Misra A.N., Mittler R., Shintani D. (2009). Thiamin confers enhanced tolerance to oxidative stress in Arabidopsis. Plant Physiol..

[56] Tian S., Wang D., Yang L., Zhang Z., Liu Y. (2022). A systematic review of 1-Deoxy-D-xylulose-5-phosphate synthase in terpenoid biosynthesis in plants. Plant Growth Regul..

[57] Battilana J., Costantini L., Emanuelli F., Sevini F., Segala C., Moser S., Velasco R., Versini G., Grando M.S. (2009). The 1-deoxy-d-xylulose 5-phosphate synthase gene co-localizes with a major QTL affecting monoterpene content in grapevine. Theor. Appl. Genet..

[58] Lange B.M., Ghassemian M. (2003). Genome organization in *Arabidopsis thaliana*: A survey for genes involved in isoprenoid and chlorophyll metabolism. Plant Mol. Biol..

[59] Zhang S., Ding G., He W., Liu K., Luo Y., Tang J., He N. (2020). Functional Characterization of the *1-Deoxy-D-Xylulose 5-Phosphate Synthase Genes* in *Morus notabilis*. Front. Plant Sci..

[60] Kambampati R., Lauhon C.T. (2000). Evidence for the Transfer of Sulfane Sulfur from IscS to ThiI during the in vitro Biosynthesis of 4-Thiouridine in *Escherichia coli* tRNA. J. Biol. Chem..

[61] Larsson O., Wahlestedt C., Timmons J.A. (2005). Considerations when using the significance analysis of microarrays (SAM) algorithm. BMC Bioinform..

[62] Löffler-Wirth H., Kalcher M., Binder H. (2015). oposSOM: R-package for high-dimensional portraying of genome-wide expression landscapes on bioconductor. Bioinformatics.

[63] Kohonen T. (1982). Self-organized formation of topologically correct feature maps. Biol. Cybern..

[64] Wirth H., Bergen M., von Binder H. (2012). Mining SOM expression portraits: Feature selection and integrating concepts of molecular function. BioData Min..

[65] Hopp L., Wirth H., Fasold M., Binder H. (2013). Portraying the expression landscapes of cancer subtypes: A case study of glioblastoma multiforme and prostate cancer. Syst. Biomed..

[66] Loeffler-Wirth H., Kreuz M., Hopp L., Arakelyan A., Haake A., Cogliatti S.B., Feller A.C., Hansmann M.-L., Lenze D., Möller P. (2019). A modular transcriptome map of mature B cell lymphomas. Genome Med..

[67] Prieto J.A., Lebon É., Ojeda H. (2010). Stomatal behavior of different grapevine cultivars in response to soil water status and air water vapor pressure deficit. OENO One.

[68] Coupel-Ledru A., Lebon É., Christophe A., Doligez A., Cabrera-Bosquet L., Péchier P., Hamard P., This P., Simonneau T. (2014). Genetic variation in a grapevine progeny (*Vitis vinifera* L. cvs Grenache×Syrah) reveals inconsistencies between maintenance of daytime leaf water potential and response of transpiration rate under drought. J. Exp. Bot..

[69] Coupel-Ledru A., Tyerman S.D., Masclef D., Lebon E., Christophe A., Edwards E.J., Simonneau T. (2017). Abscisic Acid Down-Regulates Hydraulic Conductance of Grapevine Leaves in Isohydric Genotypes Only. Plant Physiol..

[70] Zhang W., Wang S.-C., Li Y. (2023). Molecular mechanism of thiamine in mitigating drought stress in Chinese wingnut (*Pterocarya stenoptera*): Insights from transcriptomics. Ecotoxicol. Environ. Saf..

[71] Rapala-Kozik M., Wolak N., Kujda M., Banas A.K. (2012). The upregulation of thiamine (vitamin B1) biosynthesis in *Arabidopsis thaliana* seedlings under salt and osmotic stress conditions is mediated by abscisic acid at the early stages of this stress response. BMC Plant Biol..

[72] Ye X.-F., Li Y., Liu H.-L., He Y.-X. (2020). Physiological analysis and transcriptome sequencing reveal the effects of drier air humidity stress on *Pterocarya stenoptera*. Genomics.

[73] Li Y., Si Y.-T., He Y.-X., Li J.-X. (2021). Comparative analysis of drought-responsive and -adaptive genes in Chinese wingnut (*Pterocarya stenoptera* C. DC). BMC Genomics.

[74] Kinsella R.J., Kähäri A., Haider S., Zamora J., Proctor G., Spudich G., Almeida-King J., Staines D., Derwent P., Kerhornou A. (2011). Ensembl BioMarts: A hub for data retrieval across taxonomic space. Database.

[75] Yates A.D., Allen J., Amode R.M., Azov A.G., Barba M., Becerra A., Bhai J., I Campbell L., Martinez M.C., Chakiachvili M. (2022). Ensembl Genomes 2022: An expanding genome resource for non-vertebrates. Nucleic Acids Res..

[76] Kanehisa M. (2000). KEGG: Kyoto Encyclopedia of Genes and Genomes. Nucleic Acids Res..

[77] Grimplet J., Cramer G.R., Dickerson J.A., Mathiason K., Hemert J.V., Fennell A.Y. (2009). VitisNet: “Omics” Integration through Grapevine Molecular Networks. PLoS ONE.

[78] Thomas P.D., Ebert D., Muruganujan A., Mushayahama T., Albou L.-P., Mi H. (2022). PANTHER: Making genome-scale phylogenetics accessible to all. Protein Sci..

